# Exploring Ethnobotany in the Catalan Linguistic Area: Traditional Plant-Based Knowledge for Addressing Gastrointestinal, Metabolic, and Nutritional Disorders

**DOI:** 10.3390/plants13172453

**Published:** 2024-09-02

**Authors:** Fuencisla Cáceres, Joan Vallès, Airy Gras

**Affiliations:** 1 Laboratori de Botànica–Unitat Associada CSIC, Facultat de Farmàcia i Ciències de l’Alimentació–Institut de Recerca de la Biodiversitat IRBio, Universitat de Barcelona, 08028 Barcelona, Catalonia, Spain; 2 Secció de Ciències Biològiques, Institut d’Estudis Catalans, 08001 Barcelona, Catalonia, Spain

**Keywords:** ethnopharmacology, folk remedies, Iberian Peninsula, medicinal plants, Mediterranean, traditional knowledge

## Abstract

Ethnobotanical research in the Catalan linguistic area (CLA) is crucial due to the persistence of traditional medicinal plant knowledge. Gastrointestinal, metabolic, and nutritional disorders are major global health issues requiring effective treatments. This study aimed to analyze plants used for these disorders in the CLA, compare the findings with phytotherapy literature, and examine correlations between plant use in humans and animals. Data were sourced from the database of the research group of Catalan ethnobotany at the University of Barcelona and the Botanic Institute of Barcelona, representing a collection of ethnobotanical studies. A total of 630 plant taxa were examined, with 15,252 use reports (UR) provided by 2301 informants. Gastrointestinal disorders were the focus, comprising 94.24% of the UR. The high informant consensus factor (0.96) indicated strong reliability of the results. The most often reported species were *Matricaria recutita* (5.97%), *Thymus vulgaris* (5.12%), and *Lippia triphylla* (4.90%). Lamiaceae (19.86%), Asteraceae (18.78%), and Rosaceae (5.55%) were the top botanical families. The main uses were digestive (17.62%), intestinal anti-inflammatory (15.68%), and antidiarrheal (8.91%). The main part of the plant used was the aerial part (33.22%). Tisane (69.25%) was the most cited pharmaceutical form. Traditional uses were confirmed by pharmacological literature in 57.59% of cases. This work highlights the potential of medicinal plants for treating gastrointestinal, metabolic, and nutritional disorders in the CLA. Further research is possible in other territories, as well as in the phytochemical and medicinal aspects of the plants recorded.

## 1. Introduction

Apart from preserving the popular knowledge of medicinal plants, ethnobotany plays a crucial role in the search for new drugs [1,2]. Today, the scope of ethnobotany has expanded, encompassing industrialized societies where the relationship between humans and their botanical environment is still significant. In Europe, the Catalan linguistic area (CLA) is one of the most extensively studied regions from an ethnobotanical perspective [3].

The World Health Organization (WHO) highlights that traditional and complementary medicines are significant yet often underestimated health resources. In fact, 88% of WHO member states recognize the use of traditional and complementary medicine, in which the majority of traditional therapies involve herbal medicines [4].

Gastrointestinal disorders, which affect approximately 60–90% of the population, represent one of the most critical systems in biological processes [5]. Many of these disorders are difficult to diagnose and treat effectively due to the symptomatology associated, which is common, unpleasant and complex, necessitating the development of novel therapies [6]. Concurrently, the incidence of metabolic disorders, which are caused by disruptions in normal metabolic processes either congenitally or through acquisition, is rapidly increasing worldwide [7]. Lastly, a balanced diet is vital for energy, growth, repair, and regulation of important bodily functions [8], with vitamins and minerals being essential components in many biochemical pathways and enzymatic processes. Nutritional imbalances, whether due to excess or deficiency, can lead to various disease states [9].

Only in Catalonia, the most populated region within the CLA, there were 3302 deaths (4.82% of total deaths) due to gastrointestinal disorders and 2377 deaths (3.47% of total deaths) due to metabolic and nutritional disorders in 2021. Overall, these disorders ranked among the top ten causes of death [10].

The principal objectives of this meta-analysis regarding the treatment of gastrointestinal, metabolic, and nutritional disorders in the CLA are as follows: (i) to evaluate and analyze the different ethnobotanical data collected; (ii) to review the findings in the context of the phytotherapeutic literature to estimate the potential of the uses from the most cited species; (iii) to assess a correlation between the plants used to treat humans and animals; and (iv) to provide evidence of the importance of traditional knowledge as a resourceful tool in the search for new plant-based drugs.

## 2. Materials and Methods

### 2.1. Study Area

**Figure 1:**
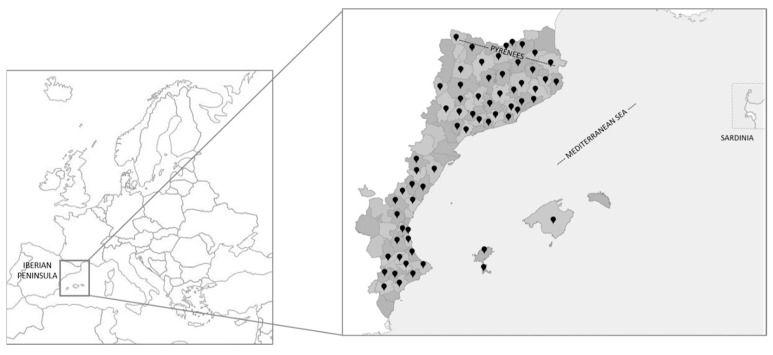
Map of the CLA in the European context. Dots represent the territories where the data were extracted. The subdivisions in the continental part correspond to administrative districts (called ‘comarca’, pl. ‘comarques’, in Catalan).

The Catalan linguistic area (CLA), also referred to as the Catalan-speaking area, Catalan-language territories, or Catalan countries, is a well-studied region from various perspectives, such as geographic [11], physiographic [12], floristic [13], vegetation [14], linguistic, and cultural [15]. This area, principally located in the eastern section of the Iberian Peninsula, also comprises the Balearic Islands, a portion of the northern Pyrenees, and the city of l’Alguer on the island of Sardinia. Politically, the CLA belongs to four states: Andorra (the entire territory), France (Northern Catalonia or Eastern Pyrenees department), Italy (l’Alguer, Sardinia), and Spain (Balearic Islands, Carxe, a small area in Murcia, Catalonia, a portion of eastern Aragon, and Valencia). These territories cover approximately 70,000 km^2^ [13] and had a population of around 14,600,000 inhabitants in 2023 [16,17,18,19]. This region extends from the level of the Mediterranean Sea to 3143 m a.s.l. in the Pyrenees at Pica d’Estats. The diverse landscapes result from different floristic and vegetative features [13,14]. These landscapes support about 4300 autochthonous and 1200 allochthonous plant taxa, including species and subspecies [20]. In this particular meta-analysis, the data were collected from 56 territories across the CLA, which are represented with dots in Figure 1.

### 2.2. Previous Fieldwork, Botanical Identification, and Databasing

The ethnobotanical information was collected through semi-structured ethnobotanical interviews [21] following the ethical principles of the International Society of Ethnobiology [22]. Then, the data extracted from these interviews were compiled in the database of the research group of the Catalan ethnobotany of the University of Barcelona and the Botanic Institute of Barcelona (http://www.etnobiofic.cat, accessed on 23 April 2024); a part of these data is available on the open-access webpage (https://etnobotanica.iec.cat, accessed on 23 April 2024) [23]. This comprehensive database is composed of numerous studies conducted between 1990 and 2024.

Medicinal uses were classified, according to Cook [24], with minor modifications. The specimens cited in each ethnobotanical interview were recollected and then deposited in the herbarium BCN (Centre de Documentació de Biodiversitat Vegetal, Universitat de Barcelona). For taxa nomenclature, we followed Bolòs et al.’s criteria [13], since this work is specific to the flora of the CLA, and Plants of the World Online [25] was used for the exotic plants. For the classification of botanical families, the latest version of the Angiosperm Phylogeny Group (APG IV) [26] was followed.

### 2.3. Data Analysis

For this meta-analysis, data were extracted from the database mentioned before, with a particular emphasis on use reports (hereafter referred to as UR), which is an input about how a person uses a specific plant for a medicinal purpose, related to gastrointestinal, metabolic, and nutritional disorders. To be more precise, each UR, cited for one informant, refers simultaneously to the plant, the specified part of the plant utilized, the specific use, and the pharmaceutical form applied. Any variation of informants, plant taxon part used, or pharmaceutical form contributes to a new UR. Data analysis was performed using the RStudio program [27] and its R packages, including tidyverse [28], plotrix [29], and ethnobotanyR [30] for both qualitative and quantitative analysis, complemented with illustrative graphics.

To evaluate the overall state of knowledge, several ethnobotanical indices were calculated. Firstly, the informant consensus factor (F_IC_) was employed to gauge the consistency and robustness of traditional knowledge regarding gastrointestinal, metabolic, and nutritional disorders in the CLA. The formula is as follows: F_IC_ = (UR − n)/(UR − 1), where UR represents the total number of use reports and (n) denotes the total number of plant taxa used. The maximum value of this parameter is one [31]. Additionally, the ethnobotanicity index (EI) was computed, representing the quotient of the total number of plant taxa utilized for gastrointestinal, metabolic, and nutritional disorders (n) and the total number of plants in the area (N), expressed as a percentage. In both parameters (n and N), native and non-native plants were considered, as they are established in the area. The equation is as follows: EI = (n/N) × 100. This index offers an insight into the significance of these plants within the region [32]. Furthermore, another index concerning individual species was established; for instance, the cultural importance (CI) index was employed to identify the most valued plants by informants, calculated as the sum of the proportion of informants mentioning each taxon’s use: CI = ΣuNCu = u1 ΣiNi = i1 URui/n [33]. Finally, the medicinal importance (MI) index was determined, indicating the importance of each specific use. It represents the ratio of the total number of UR for a specific use category to the total number of plant taxa possessing this use (n): MI = UR/n [34]. For all these calculations, species identified only at the genus level were excluded to ensure accuracy and specificity in the data analysis.

### 2.4. Comparison with Phytotherapy and Pharmacology Sources

A pharmacological comparison was accomplished by checking the uses of the most reported plant taxa with the activity associated to them in a pool of renowned encyclopedic literature on phytotherapy [35,36], databases [37,38,39] and monographs from official sources [40,41,42,43,44,45,46]. The purpose of this comparison is to analyze the number of uses with phytochemical and or pharmacological information and to assemble evidence of two scientific approaches: ethnobotanical and phytotherapeutic/pharmacological.

## 3. Results and Discussion

### 3.1. General Data

In this extensive analysis of gastrointestinal, metabolic and nutritional disorders, a total of 15,252 UR were provided by 2301 informants. Among the 630 taxa concerned, 22 were classified solely at the generic level and 59 at the infraspecific level, all of them were distributed across 104 botanical families.

The majority of the UR were associated with the treatment of gastrointestinal disorders, consisting of 14,374 UR (94.24%). In contrast, metabolic disorders accounted for 754 UR (4.94%), and nutritional disorders were represented by 124 UR (0.81%). The statistics elucidate the pronounced emphasis on gastrointestinal disorders within this study, indicative of their pervasive prevalence and significant efforts to mitigate the symptomatic burden they impose, often manifesting as highly discomforting and troublesome for the afflicted individuals. This emphasis is consistent with the traditional medicinal approach, which typically targets mild symptoms, although it should not be overlooked.

The overall F_IC_ was 0.96, nearly reaching its maximum possible value, underscoring the consistency, robustness and reliability of the collected data. The F_IC_ calculated for gastrointestinal, metabolic and nutritional disorders exceeds the F_IC_ values reported for various other ailments in other meta-analyses within the CLA, such as infectious diseases (0.92) [47], and mental disorders (0.93) [48]. Additionally, it is higher than the F_IC_ values from meta-analyses in the Ripollès district, which is also part of the CLA, for topical uses (0.93) [49] and for respiratory tract infections (0.83) [50]. This fact indicates a strong consensus among informants regarding the efficacy of the identified medicinal plant for gastrointestinal, metabolic and nutritional disorders.

It is noteworthy that this study investigates three distinct disorders, whereas previous research typically focused on one particular disorder. Specifically, the F_IC_ values for each disorder were 0.96 for gastrointestinal disorders, 0.80 for metabolic disorders, and 0.42 for nutritional disorders. These values advocate varying degrees of consensus and knowledge among informants about the different types of disorders, with gastrointestinal disorders being the most well-understood and widely addressed in traditional practices.

Furthermore, the proportion of useful flora in the region, evaluated for the treatment of gastrointestinal, metabolic, and nutritional disorders, as indicated by the EI, was notably high, at 10.16%. This value surpasses the EI calculated for other ailments in the CLA, such as infectious diseases (7.26%) [47] and mental disorders (3.72%) [48], suggesting a greater diversity of plant use among informants in addressing the disorders examined in this survey.

### 3.2. Taxa

Among the 630 taxa analyzed, Table 1 meticulously presents the 15 taxa with the highest frequency, which collectively constitute 40.18% of the total reports analyzed. The selection was made based on the observation that taxa below this threshold accounted for less than 1% of the total UR.

Moreover, Table 1 offers an overview of the statistics for the top fifteen species, enabling a deeper analysis through comparisons with existing phytotherapy bibliography. Given the bioecological and cultural similarities across the Iberian Peninsula and the Mediterranean basin, it is valuable to compare findings. Studies from various countries report the following number of taxa used: Spain (126 taxa) [51], Türkiye (33 taxa) [52], Algeria (50 taxa [53]; 33 taxa [54]), and Morocco (216 taxa) [55]. Notably, this study documents a significantly higher number of taxa, with 630 taxa identified, highlighting the extensive diversity and richness of traditional medicinal knowledge regarding gastrointestinal disorders in the CLA.

The aforementioned surveys focus exclusively on gastrointestinal disorders. Idm’hand et al. [55] conducted a recent comprehensive review of gastrointestinal disorders in Morocco, incorporating nearly all pertinent ethnobotanical works available at the time. Therefore, other surveys on gastrointestinal diseases in Morocco were not considered. Unfortunately, a study on metabolic disorders performed in Morocco [56] did not identify any of the species among the top 15 analyzed in this work. In addition, the search for surveys on nutritional disorders in the Mediterranean region was unsuccessful.

**Figure 2:**
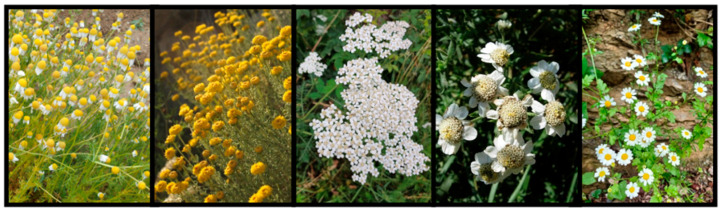
Pictures of the plants called ‘camamilla’. From left to right: *Matricaria recutita* L., *Santolina chamaecyparissus* L., *Achillea millefolium* L., *Achillea ptarmica* L. subsp. *pyrenaica* (Godr.) Heimerl., and *Tanacetum parthenium* (L.) Sch.Bip.

Interestingly, informants referred to *Matricaria recutita* L., *Santolina chamaecyparissus* L., *Achillea millefolium* L., *Achillea ptarmica* L. subsp. *pyrenaica* (Godr.) Heimerl., and *Tanacetum parthenium* (L.) Sch.Bip. by the same Catalan name, ‘camamilla’. These species not only exhibit visual similarities (Figure 2), but also belong to the same botanical family, Asteraceae. Additionally, they were cited for addressing similar gastrointestinal disorders, indicating a clear trend (see the “Ethnobotanical Uses” section). They are not merged or indistinguishable by the informants, as some of them are often specifically identified by the names (‘camamilla de muntanya’ or ‘camamilla de botó’, for instance); however, ‘camamilla’ constitutes a relevant supraspecific and suprageneric ethnotaxon, importantly associated to digestive ailments.

In this study, *Matricaria recutita* L. demonstrated the highest CI (0.40), followed by *Thymus vulgaris* L. (0.34) and *Lippia triphylla* (L’Hér.) O.Kuntze (0.33). Similarly, other higher-ranked taxa showed elevated CI values (see Table S1 in Supplementary Materials for specifics), reflecting that most cited plants were perceived with efficacy and as culturally relevant by the informants.

Remarkably, one-third of the top fifteen plants most reported in this analysis for their efficacy in treating gastrointestinal, metabolic and nutritional disorders (Table 1) are concurrently acknowledged as folk functional foods [57]. The species *Thymus vulgaris* L., *Sambucus nigra* L., *Gentiana lutea* L., *Mentha spicata* L. and *Mentha pulegium* L. have demonstrated their dual utility in serving both medicinal and nutritional functions.

**Table 1:** The most cited plants for treating gastrointestinal, metabolic, and nutritional disorders, along with their accepted names [25] (where applicable), traditional local names, uses, total use reports, percentages, CI index, and other recorded gastrointestinal uses from Mediterranean areas, were analyzed. A comparison of these uses with those documented in the pharmacological literature was conducted, which corresponded to ^1^ Blumenthal [36]; ^2^ Handbook of 200 Medicinal Plants [35]; ^3^ Dr. Duke’s Phytochemical and Ethnobotanical Databases [38]; ^4^ Fitoterapia.net [39]; ^5^ Plants For A Future [37]; ^6^ European Union Herbal Monographs [41]; ^7^ ESCOP Herbal Monographs [42]; ^8^ The Complete German Commission E monographs [40]; and ^9^ WHO monographs on selected medicinal plants [43,44,45,46].

Taxon (Accepted Name [25]), Family, Herbarium Voucher	Traditional Local Names (in Catalan Language)	Uses and Pharmacological Comparison	UR	UR (%)	CI	Recorded Gastrointestinal Uses from Mediterranean Areas
*Matricaria recutita* L. (*Matricaria chamomilla* L.), Asteraceae, BCN 140183	Camamilla, camamilla dolça, camamilla fina, mançanilla	Antidiarrheal ^1,2,3,7,9^, antiemetic, antinauseous ^4,7^, antiulcerous ^2,4,5,6,7^, carminative ^1,2,3,4,6,5,7,9^, digestive ^1,2,3,4,9^, emetic ^5^, for colic ^1,2,3,4,5,6,7,8^, for dyspepsia ^2,9^, for gastrointestinal disorders ^1,2,4,5,6,7,8,9^, for toothache ^5^, gastric anti-inflammatory ^1,2,3,4,5,7,8^, gastric/intestinal emollient ^3^, gingival anti-inflammatory ^1,2,3,4,5,6,7,8,9^, hepatoprotective, intestinal anti-inflammatory ^1,2,3,4,5,7,8^, laxative ^2^, orexigenic, purgative, refrigerant, stomachic ^2,3,5^	911	5.97	0.40	Evil ache, digestive, stomach ache [51]. Treatment of gastrointestinal disorders [52]. Indigestion, abdominal pain [55].
*Thymus vulgaris* L., Lamiaceae, BCN 25023	Farigola, frígola, farigoleta, tem, timó, timonet, tomanil, tremoncell	Antacid, antidiarrheal ^2,5^, antiemetic, anti-icteric, antinauseous, antiulcerous, buccal anti-inflammatory ^2,3,4,5^, buccal antiseptic ^2,3,4,5,9^, carminative ^2,3,5^, digestive ^2,3,4,5^, for dental reinforcement ^9^, for dyspepsia ^2,9^, for gastrointestinal disorders ^9^, for toothache, gastric anti-inflammatory ^2,4,5^, gingival anti-inflammatory ^2,4,9^, gingival antiseptic ^2,3,4,5,9^, hepatic anti-inflammatory ^2,4^, hepatic lithotriptic ^2^, hepatoprotective, hypolipemic, intestinal anti-inflammatory ^2,4^, laxative, orexigenic ^2,3,4^, purgative, refrigerant, stomachic	781	5.12	0.34	Digestive [51]. Acute ache, digestion problems, intestinal comfort, gastric ulcer [55].
*Lippia triphylla* (L’Hér.) O.Kuntze (*Aloysia citrodora* Paláu), Verbenaceae, BCN 125394	Herballuïsa, marialluïsa	Antacid ^5^, antidiarrheal ^3^, antiemetic, antinauseous ^3^, buccal antiseptic, carminative ^4,5,6^, digestive ^4,5^, for dyspepsia ^4^, for gastrointestinal disorders ^4,5,6^, for toothache, gastric anti-inflammatory ^3,4^, hepatoprotective, hypouricemic, intestinal anti-inflammatory ^4^, laxative, orexigenic, refrigerant, stomachic ^5^	747	4.90	0.33	Intestinal comfort, intestinal diseases, dyspepsia [55]. Treatment of gastrointestinal disorders [53].
*Santolina chamaecyparissus* L., Asteraceae, BCN 96763	Botja, camamilla, camamilla de botó, camamilla de muntanya, camamilla de Maó, camamil·la, camamirla, espernallac, espernellac	Antidiarrheal, antiemetic, anti-icteric, antinauseous, antiulcerous ^4^, buccal anti-inflammatory ^4^, buccal antiseptic ^4,5^, carminative ^4^, digestive ^3,4,5^, for gastrointestinal disorders ^4,5^, for toothache, gastric anti-inflammatory ^4^, gingival anti-inflammatory ^4^, hepatic anti-inflammatory ^4^, hepatoprotective, intestinal anti-inflammatory ^4^, laxative, orexigenic ^4^, purgative, refrigerant, stomachic ^3^	500	3.28	0.22	Digestive [51]. Against indigestion and gastralgia [53].
*Anemone hepatica* L., (*Hepatica nobilis* Schereb.), Ranunculaceae, BCN 27247	Fetgera, herba fetgera, herba del fetge, viola de llop	Antiemetic, anti-icteric, carminative, digestive ^5^, for gastrointestinal disorders, hepatic anti-inflammatory ^3^, hepatic lithotriptic, hepatoprotective ^4,5^, intestinal anti-inflammatory ^4^, laxative	450	2.95	0.20	-
*Mentha pulegium* L., Lamiaceae, BCN 113598	Menta, poliol, poniol, poriol	Antidiarrheal, antiemetic, antinauseous, carminative ^3,4,5^, digestive ^3,4,5^, gastric anti-inflammatory, hepatic anti-inflammatory, hepatoprotective, intestinal anti-inflammatory, orexigenic ^4^, refrigerant, stomachic ^3^	369	2.42	0.16	Acute ache, digestion problems, intestinal comfort, dyspepsia/indigestion, abdominal pain [55]. Colon ailments, stomach ache [54].
*Gentiana lutea* L., Gentianaceae, BCN 24893	Genciana, gençana, gençana groga, gençana negra, herba gençana, llenciana, llençana	Buccal antiseptic ^2^, digestive ^2,4,5,6,8^, for appendicitis, for toothache, hepatoprotective ^2,5^, hypolipemic, intestinal anti-inflammatory ^2,5,7,9^, laxative ^9^, orexigenic ^2,3,4,5,6,7,8,9^, purgative, stomachic ^2,3,5,6^	367	2.41	0.16	-
*Sambucus nigra* L., Adoxaceae, BCN 24984	Benabre, sabuc, sabuquer, saüc, saüquer, saüguer, suguer	Antidiarrheal ^5,9^, antiemetic, antinauseous, antiulcerous, buccal antiseptic ^3^, carminative, diaphoretic ^3,4,5,7,8,9^, digestive, for colic ^5^, for dyspepsia, for gastrointestinal disorders, for metabolic disorders, for toothache, gastric anti-inflammatory ^7,9^, hepatic anti-inflammatory ^7,9^, hepatoprotective, hypolipemic, hypouricemic, intestinal anti-inflammatory ^7,9^, laxative ^3,4,5,9^, orexigenic ^3^, purgative ^3,5^, refrigerant, stomachic ^3^	361	2.37	0.16	Phlegmon, toothache [51].
*Foeniculum vulgare* Mill. subsp. *piperitum* (Ucria) Cout., Apiaceae, BCN 125404	Fenoll, fonoll	Antidiarrheal ^2,4,9^, antiemetic ^2,4^, antihalitosic ^5^, buccal anti-inflammatory ^4,5,6,9^, buccal antiseptic ^4,5^, carminative ^2,3,4,5,6,7,8,9^, digestive ^2,3,4,5,8^, for colic ^2,3,4,5,6,7,8^, for gastrointestinal disorders ^2,3,4,5,6,8^, for obesity treatment, gastric anti-inflammatory ^2,3,4,5,6,9^, hepatoprotective ^7^, hypolipemic, intestinal anti-inflammatory ^2,3,4,5,6^, laxative ^2,4,5,9^, orexigenic ^4,9^, refrigerant, sialagogic, stomachic ^2,3,5,7^, vitaminic	320	2.10	0.14	Digestive, stomach acidity, stomach ache, tonic, antispasmodic, carminative, intestinal problems, mouth injuries [51]. Stomach pain, acute ache, digestion problems, intestinal comfort, bloating, intestinal gas, anorexia, intestinal diseases, flatulence, belching, irritable bowel syndrome, mouth, tongue, lip symptom, swallowing problem [55]. Against indigestion and gastralgia [53].
*Achillea millefolium* L., Asteraceae, BCN 125391	Camamilla borda, herba de cent fulles, herba de les cent fules, herba de mil fulles, lladracà, milfulles, milifulla,	Antacid ^2^, antidiarrheal ^2,5^, antiemetic, antinauseous, antiulcerous ^2,4^, buccal antiseptic ^2,3,5^, digestive ^2,5^, emetic, for colic ^2,4,5,6,8^, for dyspepsia ^3,8,9^, for gastrointestinal disorders ^2,6,8,9^, gastric anti-inflammatory ^2,3,4,5,6,9^, hepatic anti-inflammatory ^2,4,5,6,9^, hepatoprotective ^2^, hypolipemic, hypouricemic, intestinal anti-inflammatory ^2,3,4,5,6,9^, laxative ^2,9^, orexigenic ^2,3,4,5,6,8,9^, purgative, refrigerant, stomachic ^3^, vitaminic	304	1.99	0.13	Digestive, stomach ache, evil tripe [51]. Gastric disorders, anorexia [55].
*Malva sylvestris* L., Malvaceae, BCN 125508	Malva, mauva, vauma	Antidiarrheal, antiulcerous ^2^, buccal anti-inflammatory ^2,3,4,6,7,8^, buccal antiseptic ^3^, diaphoretic, digestive ^5^, for gastrointestinal disorders ^2,3,4,5,6^, for toothache ^3^, gastric anti-inflammatory ^2,3,4,6^, gastric/intestinal emollient ^2,3,4,5,6,7,8^, gingival anti-inflammatory ^2,3,4,6,7,8^, gingival antiseptic ^3^, hepatic anti-inflammatory ^2,3,4^, hepatoprotective, intestinal anti-inflammatory ^2,3,4,6^, laxative ^2,4,5,6^, purgative, refrigerant ^3^, stomachic	265	1.74	0.12	Digestive [51]. Against gastralgia, laxative [53]. Diarrhea [55]. Against gastralgia, laxative [52].
*Mentha spicata* L., Lamiaceae, BCN 24930	Herba-sana, menta, menta del consol, menta de les faves, menta de la sopa	Antidiarrheal ^3^, antiemetic ^5^, antinauseous ^3^, buccal antiseptic ^5^, digestive, for gallbladder inflammation, for toothache, gastric anti-inflammatory, hepatic anti-inflammatory, hepatoprotective, intestinal anti-inflammatory, laxative, purgative, refrigerant, stomachic ^3,5^	233	1.53	0.10	Digestive [51]. Worms, other parasites [55]. Carminative, against gastralgia [52].
*Centaurium erythraea* Rafn, Gentianaceae, BCN 24746	Centaura, herba de Santa Aura, Santa Aura	Antidiarrheal, antiemetic, antinauseous, digestive ^5,6^, emetic ^5^, for dyspepsia ^4,5,7^, gastric anti-inflammatory ^4,6,7^, hepatic anti-inflammatory ^4,5,7^, hepatoprotective ^7^, intestinal anti-inflammatory ^4,6,7^, laxative, orexigenic ^4,5,6,7,8^, purgative, stomachic ^5^	210	1.38	0.09	Loss of appetite [51]. Against diarrhea [53]. Abdominal pain [55].
*Achillea ptarmica* L. subsp. *pyrenaica* (Godr.) Heimerl., Asteraceae, BCN 24701	Camamilla, camamilla de muntanya, camamilla de Núria, camamilla salvatge	Antidiarrheal ^5^, antinauseous, buccal antiseptic, carminative ^5^, digestive ^5^, emetic, for gastrointestinal disorders, gastric anti-inflammatory, hepatoprotective, intestinal anti-inflammatory, stomachic	157	1.03	0.07	-
*Tanacetum parthenium* (L.) Sch.Bip., Asteraceae, BCN 25014	Camamilla, camamilla borda, camamilla amarga, tanarida	Antidiarrheal ^9^, antiemetic ^1^, antinauseous ^1^, digestive ^9^, emetic, for gastrointestinal disorders ^5^, hepatoprotective, intestinal anti-inflammatory ^1,5,7,9^, laxative, purgative, stomachic ^5^	154	1.01	0.07	Digestive [51].

### 3.3. Botanical Families

**Figure 3:**
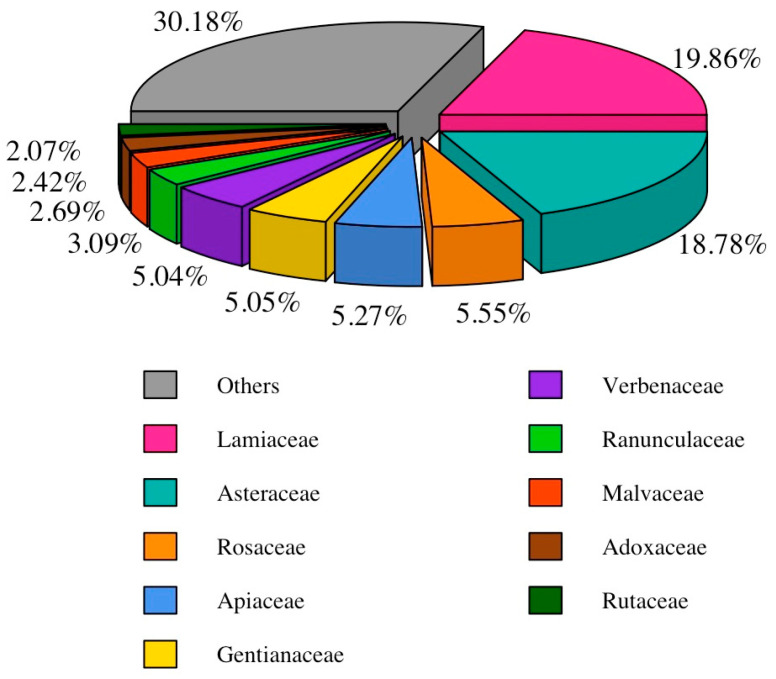
Proportion of the botanical families. ‘Others’ include all the botanical families with less than 2% of use reports.

The 10 most often reported families, which collectively represent 69.82% of the total of reports, are notable for their significant contribution to the study (see Figure 3). Lamiaceae (96 taxa; 19.86%) and Asteraceae (92 taxa, 18.78%) emerge as the two most cited families. The prevalence of these families can be attributed either to their vast diversity in species or to their widespread distribution throughout the Mediterranean landscape [25]. Of particular significance is also the prominent ranking of Lamiaceae in the treatment of gastrointestinal disorders across various Mediterranean areas [51,52,53,54,55], accentuating its pivotal role in the area’s traditional herbal medicine practices. The results contribute to the hypothesis that the Lamiaceae family is essential to promoting the overall health and well-being of the people and culture of the Mediterranean region, thanks to its well-known antioxidant, antimicrobial, and anti-inflammatory properties [58].

### 3.4. Parts of the Plant Used

**Figure 4:**
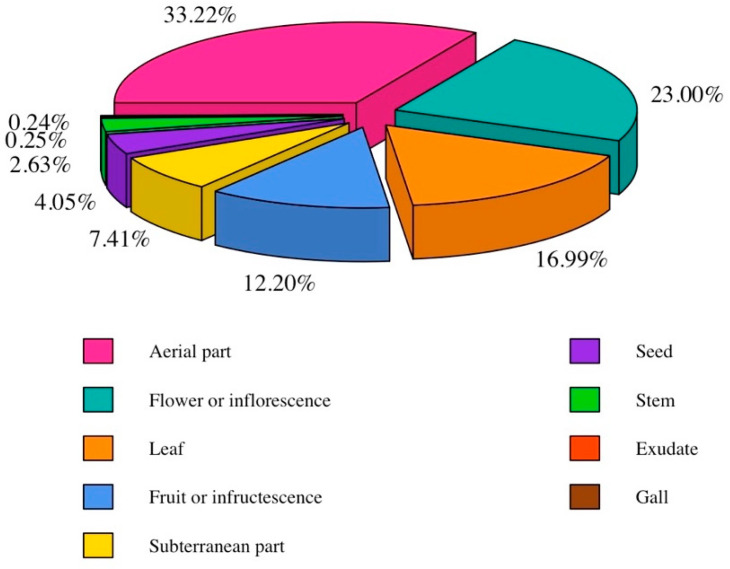
Proportion of the parts of the plants used.

To facilitate the analysis, various specific parts of the cited plants were categorized into major groups; for example, within the flower or inflorescence group, components such as sepals, petals, floral summits and other related elements were included. The subterranean part encompassed bulbs, rhizomes, tubers, and roots. Similarly, fruits or infructescence, including mesocarp, endocarp, strobilus and similar structures were grouped. The categorization was extended to other plant parts as well. Only 3.66% of the reports analyzed lacked this information; consequently, they were excluded from the analysis. Among the reported parts, the aerial part (33.22%), flower or inflorescence (23.00%), and leaf (16.99%) were the most frequently mentioned. Notably, the aerial part and flower or inflorescence were also predominantly cited parts in other ailments surveys within the CLA [47,48]. Figure 4 illustrates the proportion of these parts, regrouped into nine categories as mentioned earlier. In light of the evidence presented, the predominance of visible and easily accessible plant organs suggests that these parts are intuitive for informants to pick.

### 3.5. Pharmaceutical Forms

A similar criterion was applied to categorize pharmaceutical forms, grouping related forms into broader categories. Nonetheless, 11.99% of the reports lacked information regarding the method of administration and were consequently excluded from the analysis. Unfortunately, this percentage is relatively high, suggesting a lack of familiarity with administration methods among some informants. Of the remaining reports, 84.01% were destined for internal use, while 12.38% were intended for external use. Surprisingly, among the external pharmaceutical forms, the poultice form was used for treating intestinal inflammation, as informants believed that addressing abdominal pain externally was more effective or less invasive. Additionally, mouthwash and gargle were used as buccal antiseptics and anti-inflammatory agents, likely due to their direct application to the affected area.

Among the reported pharmaceutical forms, the most common were the following: tisane (69.25%), which enclosed different forms of preparation, such as infusion and decoction; without pharmaceutical form (11.03%) which corresponds to direct ingestion of the plant or a direct application into a part of the body without any type of previous preparation; mouthwash (4.76%); poultice (2.31%), which includes dressings and fomentations; gargle (1.32%); essence (1.23%); medicinal wine (1.19%) which incorporates medicinal vinegar and wine tincture; syrup (1.11%); and emulsion (1.02%). Other pharmaceutical forms, such as water macerate, liniment, aerosol, enema, fumigation, suspension, bath, aqueous juice, lotion, oily juice, lemonade, cream, cigarette, liquid extract, powders, candle, potion, aromatic distilled water, injectable, distilled water, drops, ointment, capsule, and dry extract, were also noted, but their presence in this analysis was less than 1%. The prevalence of the most frequent administration forms aligns with findings from meta-analytic ethnobotanical studies in the CLA [47,48].

### 3.6. Ethnobotanical Uses

In total, 46 uses were reported (see Table S2 in Supplementary Materials). Notably, 79.27% of the total UR concentrated on the tenth most cited uses. The primary uses included digestive (17.62%), intestinal anti-inflammatory (15.68%), and antidiarrheal (8.91%), highlighting a significant understanding of natural remedies for indigestion, abdominal pain, and diarrhea. Most taxa were associated with multiple uses (see the “Number of Uses” column in Table S1 of Supplementary Materials for additional details), evidencing the concept that medicinal plants often serve as multi-use species, capable of addressing various ailments.

In terms of the MI index, intestinal anti-inflammatory exhibits the highest value (57.59), followed by gastrointestinal disorders (23.43) and antinauseous (15.88). The MI index is instrumental in assessing the real importance of medicinal uses. By evaluating how a particular use is documented across a varying number of species, this indicator significantly influences the perceived relevance and reliability of information regarding the plant. In this analysis, the markedly elevated value of intestinal anti-inflammatory use (57.59) underscores the consistency in employing plants for this particular purpose.

**Figure 5:**
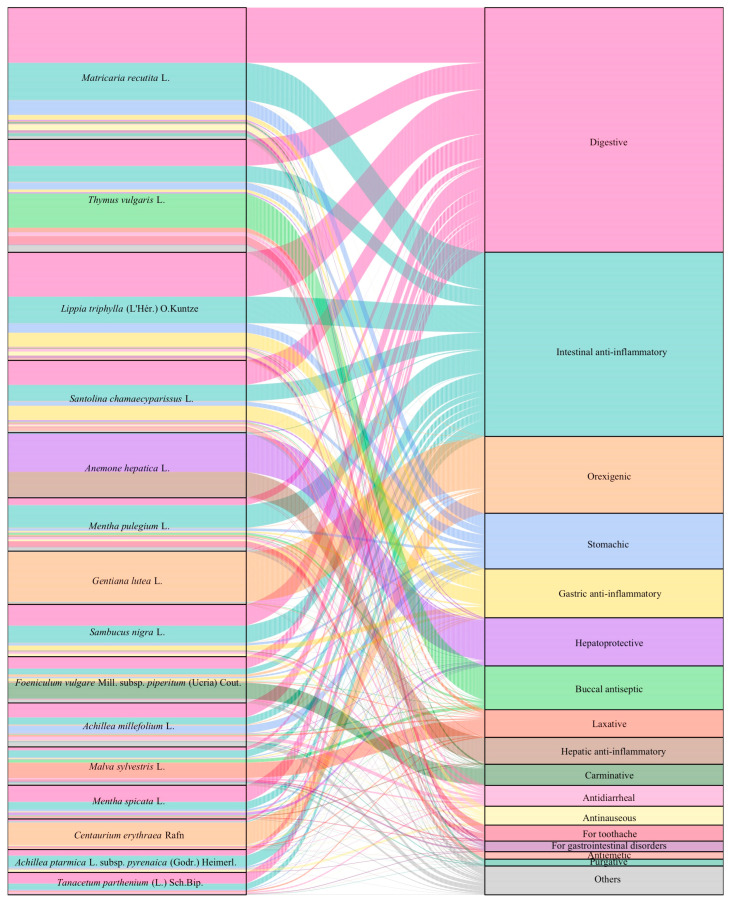
Alluvial graphic depicting the 15 most reported plant taxa and their intrinsic associations with the uses attributed to at least one of these species.

An alluvial graphic (Figure 5) was designed to illustrate the connections between the fifteen most reported taxa and their corresponding uses. To enhance clarity, a category labeled ‘Others’ was created to aggregate uses reported less frequently than 0.5%. This category includes refrigerant (0.47%), buccal anti-inflammatory (0.39%), emetic (0.36%), hypolipemic (0.33%), for dyspepsia (0.28%), antiulcerous (0.26%), gingival anti-inflammatory (0.18%), for colic (0.15%), diaphoretic (0.13%), anti-icteric (0.11%), gingival antiseptic (0.10%), antacid (0.08%), antihalitosic (0.08%), hypouricemic (0.07%), for appendicitis (0.03%), for metabolic disorders (0.03%), for obesity treatment (0.03%), gastric/intestinal emollient (0.03%), hepatic lithotriptic (0.03%), vitaminic (0.03%), for dental reinforcement (0.02%), for gallbladder inflammation (0.02%), and sialagogic (0.02%). Noteworthy utilization patterns emerge, particularly with *Matricaria recutita* L., *Lippia triphylla* (L’Hér.) O.Kuntze, *Santolina chamaecyparissus* L., *Mentha pulegium* L., *Sambucus nigra* L., *Achillea millefolium* L., *Mentha spicata* L., *Achillea ptarmica* L. subsp. *pyrenaica* (Godr.) Heimerl., and *Tanacetum parthenium* (L.) Sch.Bip., which predominantly exhibit digestive and intestinal anti-inflammatory uses. These uses are the most prevalent across the entirety of the UR analyzed, corroborating the aim for informants to address uses related to indigestion and abdominal pain due to their uncomfortable symptoms and incident recurrences.

Interestingly, despite being the third most cited use overall, antidiarrheal applications are practically absent among the top uses for these fifteen species. Nevertheless, notable exceptions deviate from the majority tendency. *Thymus vulgaris* L. demonstrates a primary use as a buccal antiseptic, aligning with its well-documented antiseptic properties [59]. *Anemone hepatica* L. presents a distinct utilization pattern, primarily serving as a hepatoprotective and hepatic anti-inflammatory agent, consistent with its scientific and vernacular names (called in Catalan ‘fetgera’ or ‘herba fetgera’, meaning ‘liver herb’) and morphology suggesting effectiveness in liver-related conditions. *Gentiana lutea* L. and *Centaurium erythraea* Rafn, both belonging to the same botanical family, differ from the norm as their applications are principally orexigenic, attributed to a shared bitter compound: gentiopicroside [60]. *Foeniculum vulgare* Mill. subsp. *piperitum* (Ucria) Cout. is particularly noticeable for being mostly used as a carminative, a well-established traditional application supported by its metabolites [61]. Lastly, *Malva sylvestris* L. exhibits a prominent use as a laxative, an extensively recorded use [62].

### 3.7. Comparison with Ethnoveterinary Data from the CLA

An intimate relationship between humans and the animal kingdom has been established since the oldest times, including animal domestication. As such, the natural remedies employed to alleviate human ailments may be used as well to treat animal diseases [63,64]. For instance, a recent study conducted in the CLA regarding gastrointestinal, metabolic and nutritional disorders [65], with 148 taxa analyzed, identified *Tanacetum parthenium* (L.) Sch.Bip., *Malva sylvestris* L., *Achillea millefolium* L., *Santolina chamaecyparissus* L., *Thymus vulgaris* L. and *Lippia triphylla* (L’Hér.) O.Kuntze (Table 1) as also being used to treat animals. Curiously, 92.57% (137 taxa out of 148) of the taxa used in ethnoveterinary were also identified in this study to treat humans.

The average UR per informant was 6.63 for human medicine, and for veterinary it was 2.35 [65]. This disparity shows that nearly everyone is familiar with plants used to treat human gastrointestinal disorders. In contrast, veterinary plant uses are more specialized, often requiring direct involvement in animal care. Other metrics such as EI and F_IC_ were lower (EI = 3.35%, F_IC_ = 0.90) [65] compared to findings in this study (EI = 10.16%, F_IC_ = 0.96), thus supporting the hypothesis that informants exhibit less familiarity with traditional veterinary knowledge.

Additionally, *Tanacetum parthenium* (L.) Sch.Bip. showed the highest CI (0.21) [65], while its CI for human use was notably lower at 0.07, highlighting reduced closeness with this plant species. Conversely, *Matricaria recutita* L., which had the highest CI (0.40), did not rank among the most cited plants in ethnoveterinary medicine. Regarding the MI index, the highest was attributed to purgative use (13.15) [65], whereas in human applications, it was notably lower at 2.45, manifesting the greater importance of this use category in veterinary contexts. Contrarily, intestinal anti-inflammatory use (57.59) was significantly more valuable in humans compared to animals (2.72) [65]. These statistics indicate that health concerns addressed in human and veterinary practices differ, thereby influencing the familiarity with selected medicinal plants used for treatment.

The aerial part, flower or inflorescence were the most reported plant parts, aligning precisely with the findings of this meta-analysis. Consequently, tisane and both internal and external direct use were the most frequently employed methods, mirroring the administration practices observed in this study. These results underline a significant consensus on the use of these plants for gastrointestinal, metabolic, and nutritional disorders, irrespective of whether the treatment is intended for humans or animals.

Given this consistency, we can affirm that there is a strong correlation between ethnobotanical and ethnoveterinary practices. The overlap in plant taxa, parts of the plant used and pharmaceutical forms, highlights a shared knowledge base and suggests that traditional medicinal practices for humans are closely intertwined with those for animals. This interrelationship further supports the importance of ethnobotanical studies in providing insights that are applicable across different contexts of medicinal use. 

### 3.8. Pharmacological Comparison

For the 15 most cited plants, an exhaustive search of the specific uses claimed by the informants in human monographs was performed from official sources, databases, and in the encyclopedic bibliography on phytotherapy (Table 1). References are quoted in the Materials and Methods section. Note that the comparison of the use is attributed with consideration to the overarching objectives of ethnobotanical research. In other words, the comparison is not solely based on the exact name match but rather on the presence of the specific illness or related activities corresponding to the ethnobotanical uses reported by our informants.

A total of 57.59% (148 out of 257) of the uses of 15 taxa considered were confirmed in the previously mentioned literature set, which represents a significant percentage. This high degree of corroboration suggests that many of the plants documented in this survey could serve as excellent candidates for alternative or complementary treatments to conventional drugs for the specific verified uses. Consequently, medicinal plants offer not only efficient therapeutic options but also cost-effective and environmentally sustainable solutions for treating these disorders. Notwithstanding, it is imperative to advocate for the rational use of flora to preserve these valuable therapeutic alternatives within the CLA.

A crucial fact to consider is that *Anemone hepatica* L., despite its traditional use for liver ailments, has not been approved for medicinal use by the Complete German Commission E monographs [40]. This is primarily due to the presence of protoanemonin, a compound that can cause severe irritations in the kidneys and urinary tract when ingested in high doses. Even so, protoanemonin is destroyed when it is dried, potentially allowing for safer use of the herb’s beneficial properties in its dried form [40].

In other Mediterranean territories, traditional knowledge has also guided people to the exploration of plant-based therapeutic options, and several of the uses reported abroad are concordant with the outcomes of this work and with the pool of literature (see Table 1 for further details). The uses cited in both Mediterranean countries and the CLA and yet not verified in phytotherapy literature were as follows: for *Thymus vulgaris* L., antiulcerous [55]; for *Mentha pulegium* L., intestinal anti-inflammatory [55] and gastric anti-inflammatory [54]; for *Sambucus nigra* L., for toothache [51]; for *Malva sylvestris* L., antidiarrheal [55]; for *Mentha spicata* L., gastric anti-inflammatory [52] and digestive [51]; for *Centaurium erythraea* Rafn, antidiarrheal [53]. Further research is needed for these traditional uses, as they present a solid promising foundation for future phytochemical and pharmacological studies.

Regrettably, various metabolic uses reported among the 15 most cited taxa were not validated in the literature, which is unfortunate because some of these uses hold potential for developing promising new treatments for major health issues. Even minor-presence uses in this survey could lead to revolutionary discoveries if further compound studies and clinical trials are conducted. A remarkable example is the case of *Foeniculum vulgare* Mill. subsp. *piperitum* (Ucria) Cout. which was noted for obesity treatments (0.62%) in this meta-analysis and, in a study performed in Korea [66], it was suggested that it may cause a short-term loss of appetite in overweight women, which can lead to an overall weight loss. Another example, *Sambucus nigra* L., was cited for metabolic disorders (0.54%), with hypouremic (0.27%), and hypolipemic properties (0.27%), an indicator of its potential in addressing these conditions, and investigations demonstrated the hypolipemic use—due to the polyphenols—in people with diabetes mellitus [67]. Regrettably, the hypouremic properties are still unstudied.

Candidates to be considered for phytochemical studies, based on the results of the present work, could be *Achillea millefolium* L. as a hypolipemic (1.64%), and *Gentiana lutea* L. as a hypolipemic (0.82%) and for appendicitis (0.52%).

## 4. Conclusions

Ethnobotanical research has paramount importance in preserving traditional knowledge and exploring alternative therapeutic options, particularly in regions like the CLA. This meta-analysis, which involved 630 taxa, underscores the significant attention given by informants to treat gastrointestinal, metabolic, and nutritional disorders, which constitute substantial health challenges globally. The high F_IC_ (0.96) reflects the robustness of the data collected, indicating a strong consensus among informants.

Moreover, the findings demonstrate that 57.59% of the reported uses were confirmed in renowned pharmacological literature, highlighting the coherent use of traditional knowledge about medicinal plants. The results obtained emphasize the idea of integrating traditional practices with modern pharmacological approaches to develop novel therapeutic interventions. It is essential to further explore the therapeutic potential of medicinal plants, especially the ones regarding gastrointestinal and metabolic disorders identified in this study, by conducting phytochemical and pharmacological studies. Additionally, this study unveils a notable correlation between plant-based remedies utilized in veterinary and human medicine, particularly in gastrointestinal, metabolic and nutritional disorders. This correlation not only underlines the synergy between traditional healing systems but also suggests extrapolating human medicinal plant uses to veterinary practices, thereby broadening therapeutic possibilities.

To summarize, this study provides valuable insights into the potential of medicinal plants in treating gastrointestinal, metabolic, and nutritional disorders. By learning from traditional knowledge and embracing interdisciplinary research, we can pave the way for the development of evidence-based therapies that benefit public health in the CLA and beyond.

## Data Availability

Data are contained within the article. The original data presented in the study are openly available in CORA (Catalan Open Research Area) at [https://doi.org/10.34810/data1495].

## Supplementary Materials

The following supporting information can be downloaded at: https://www.mdpi.com/article/10.3390/plants13172453/s1, Table S1: A comprehensive overview of all 630 plant taxa, detailing the number of use reports (UR) alongside their corresponding percentages, the cultural importance index (CI) for each species, and the total number of uses attributed to each taxon; Table S2: A representation of the 46 uses, providing details on the number of use reports (UR) and their corresponding percentages for each use, along with the total associated taxa and the medicinal importance (MI) index.
