# Supplementary material for: Exploring Ethnobotany in the Catalan Linguistic Area: Traditional Plant-Based Knowledge for Addressing Gastrointestinal, Metabolic, and Nutritional Disorders
Source: Plants (Basel). 2024 Sep 2;13(17):2453. doi: 10.3390/plants13172453 (PMC11397403; doi:10.3390/plants13172453)
Supplement: Supplementary file 1 [file plants-13-02453-s001.zip › plants-3158508-supplementary.pdf]

**Supplementary material**

**Table S1.** A comprehensive overview of all 630 plant taxa, detailing the number of use reports (UR) alongside their corresponding percentages, the cultural importance index (CI) for each species, and the total number of uses attributed to each taxon.

| Taxa | UR | UR (%) | CI | Number of uses |
|---|---|---|---|---|
| *Matricaria recutita* L. | 911 | 5.97 | 0.40 | 21 |
| *Thymus vulgaris* L. | 781 | 5.12 | 0.34 | 27 |
| *Lippia triphylla* (L'Hér.) O.Kuntze | 747 | 4.90 | 0.33 | 18 |
| *Santolina chamaecyparissus* L. | 500 | 3.28 | 0.22 | 21 |
| *Anemone hepatica* L. | 450 | 2.95 | 0.20 | 10 |
| *Sambucus nigra* L. | 369 | 2.42 | 0.16 | 24 |
| *Gentiana lutea* L. | 367 | 2.41 | 0.16 | 11 |
| *Mentha pulegium* L. | 361 | 2.37 | 0.16 | 12 |
| *Foeniculum vulgare* Mill. subsp. *piperitum* (Ucria) Cout. | 320 | 2.10 | 0.14 | 20 |
| *Achillea millefolium* L. | 304 | 1.99 | 0.13 | 23 |
| *Malva sylvestris* L. | 265 | 1.74 | 0.12 | 19 |
| *Mentha spicata* L. | 233 | 1.53 | 0.10 | 15 |
| *Centaurium erythraea* Rafn | 210 | 1.38 | 0.09 | 14 |
| *Achillea ptarmica* L. subsp. *pyrenaica* (Godr.) Heimerl. | 157 | 1.03 | 0.07 | 11 |
| *Tanacetum parthenium* (L.) Sch.Bip. | 154 | 1.01 | 0.07 | 11 |
| *Carum carvi* L. | 145 | 0.95 | 0.06 | 11 |
| *Cydonia oblonga* Mill. | 143 | 0.94 | 0.06 | 8 |
| *Ruta chalepensis* L. | 141 | 0.92 | 0.06 | 17 |
| *Hyoscyamus niger* L. | 139 | 0.91 | 0.06 | 2 |
| *Linum usitatissimum* L. | 135 | 0.89 | 0.06 | 12 |
| *Salvia officinalis* L. subsp. *lavandulifolia* (Vahl) Gams | 135 | 0.89 | 0.06 | 21 |
| *Rosmarinus officinalis* L. | 133 | 0.87 | 0.06 | 19 |
| *Cynara scolymus* L. | 130 | 0.85 | 0.06 | 11 |
| *Olea europaea* L. subsp. *europaea* var. *europaea* | 129 | 0.85 | 0.06 | 20 |
| *Euphorbia lathyris* L. | 126 | 0.83 | 0.06 | 3 |
| *Jasonia saxatilis* (Lam.) Guss. | 126 | 0.83 | 0.06 | 14 |
| *Papaver somniferum* L. | 122 | 0.80 | 0.05 | 4 |
| *Gentiana burseri* Lap. subsp. *burseri* | 120 | 0.79 | 0.05 | 4 |
| *Mentha ×piperita* L. | 120 | 0.79 | 0.05 | 14 |
| *Quercus ilex* L. | 118 | 0.77 | 0.05 | 13 |
| *Allium sativum* L. | 116 | 0.76 | 0.05 | 20 |
| *Oryza sativa* L. | 114 | 0.75 | 0.05 | 4 |
| *Herniaria glabra* L. | 113 | 0.74 | 0.05 | 10 |
| *Rubus ulmifolius* Schott | 110 | 0.72 | 0.05 | 16 |
| *Taraxacum officinale* Weber in Wiggers | 108 | 0.71 | 0.05 | 10 |
| *Celtis australis* L. | 107 | 0.70 | 0.05 | 6 |
| *Juniperus communis* L. | 106 | 0.69 | 0.05 | 11 |
| *Thymus serpyllum* L. | 102 | 0.67 | 0.04 | 17 |
| *Hyssopus officinalis* L. | 100 | 0.66 | 0.04 | 12 |
| *Mentha* sp. | 100 | 0.66 | - | 10 |
| *Ricinus communis* L. | 95 | 0.62 | 0.04 | 9 |
| *Brassica oleracea* L. subsp. *oleracea* var. *capitata* L. f. capitata | 94 | 0.62 | 0.04 | 13 |
| *Petroselinum crispum* (Mill.) Hill | 92 | 0.60 | 0.04 | 14 |
| *Juglans regia* L. | 86 | 0.56 | 0.04 | 14 |

| | | | | |
|---|---|---|---|---|
| *Allium cepa* L. | 85 | 0.56 | 0.04 | 16 |
| *Salvia officinalis* L. subsp. *officinalis* | 85 | 0.56 | 0.04 | 17 |
| *Pimpinella anisum* L. | 81 | 0.53 | 0.04 | 14 |
| *Parietaria officinalis* L. subsp. *judaica* (L.) Béguinot | 79 | 0.52 | 0.03 | 19 |
| *Melissa officinalis* L. subsp. *officinalis* | 78 | 0.51 | 0.03 | 11 |
| *Sorbus domestica* L. | 76 | 0.50 | 0.03 | 2 |
| *Pistacia lentiscus* L. | 72 | 0.47 | 0.03 | 12 |
| *Plantago major* L. | 72 | 0.47 | 0.03 | 11 |
| *Triticum aestivum* L. | 72 | 0.47 | 0.03 | 10 |
| *Citrus limon* (L.) Burm. | 69 | 0.45 | 0.03 | 20 |
| *Rosa canina* L. | 68 | 0.45 | 0.03 | 7 |
| *Polygala calcarea* F.W.Schultz | 67 | 0.44 | 0.03 | 11 |
| *Prunus domestica* L. | 66 | 0.43 | 0.03 | 3 |
| *Plantago lanceolata* L. | 65 | 0.43 | 0.03 | 13 |
| *Artemisia absinthium* L. | 64 | 0.42 | 0.03 | 15 |
| *Globularia alypum* L. | 64 | 0.42 | 0.03 | 10 |
| *Vitis vinifera* L. | 64 | 0.42 | 0.03 | 13 |
| *Satureja fruticosa* (L.) Briq. subsp. *fruticosa* | 62 | 0.41 | 0.03 | 8 |
| *Teucrium polium* L. | 62 | 0.41 | 0.03 | 15 |
| *Prunus avium* (L.) L. | 61 | 0.40 | 0.03 | 10 |
| *Origanum vulgare* L. | 60 | 0.39 | 0.03 | 8 |
| *Hyoscyamus albus* L. | 59 | 0.39 | 0.03 | 2 |
| *Lithospermum officinale* L. | 54 | 0.35 | 0.02 | 9 |
| *Cynodon dactylon* (L.) Pers. | 52 | 0.34 | 0.02 | 17 |
| *Satureja calamintha* (L.) Scheele | 52 | 0.34 | 0.02 | 7 |
| *Centaurium quadrifolium* (L.) G.López et Ch.E.Jarvis | 48 | 0.31 | 0.02 | 9 |
| *Laurus nobilis* L. | 48 | 0.31 | 0.02 | 10 |
| *Lavandula stoechas* L. | 48 | 0.31 | 0.02 | 8 |
| *Punica granatum* L. | 48 | 0.31 | 0.02 | 10 |
| *Scirpus holoschoenus* L. | 48 | 0.31 | 0.02 | 8 |
| *Sideritis angustifolia* Lag. | 47 | 0.31 | 0.02 | 12 |
| *Eryngium campestre* L. | 43 | 0.28 | 0.02 | 14 |
| *Papaver rhoeas* L. | 39 | 0.26 | 0.02 | 7 |
| *Beta vulgaris* L. subsp. *vulgaris* var. *vulgaris* | 38 | 0.25 | 0.02 | 6 |
| *Origanum majorana* L. | 38 | 0.25 | 0.02 | 7 |
| *Pyrus malus* L. | 38 | 0.25 | 0.02 | 7 |
| *Eriobotrya japonica* (Thunb.) Lindl. | 37 | 0.24 | 0.02 | 4 |
| *Coris monspeliensis* L. | 36 | 0.24 | 0.02 | 7 |
| *Crataegus monogyna* Jacq. | 36 | 0.24 | 0.02 | 7 |
| *Citrus sinensis* (L.) Osbeck | 35 | 0.23 | 0.02 | 12 |
| *Ruta graveolens* L. | 35 | 0.23 | 0.02 | 12 |
| *Peucedanum ostruthium* (L.) Koch | 34 | 0.22 | 0.02 | 8 |
| *Sideritis hyssopifolia* L. | 34 | 0.22 | 0.02 | 6 |
| *Daucus carota* L. subsp. *sativus* (Hoffm.) Arcang. | 33 | 0.22 | 0.01 | 4 |
| *Marrubium vulgare* L. | 33 | 0.22 | 0.01 | 10 |
| *Urtica dioica* L. | 33 | 0.22 | 0.01 | 16 |
| *Peumus boldus* Molina | 32 | 0.21 | 0.01 | 4 |
| *Rosa ×centifolia* L. | 31 | 0.20 | 0.01 | 3 |
| *Cassia angustifolia* Vahl. | 30 | 0.20 | 0.01 | 2 |
| *Hypericum perforatum* L. | 30 | 0.20 | 0.01 | 14 |
| *Xanthium spinosum* L. | 30 | 0.20 | 0.01 | 7 |

| | | | | |
|---|---|---|---|---|
| *Juniperus oxycedrus* L. | 29 | 0.19 | 0.01 | 11 |
| *Malva neglecta* Wallr. | 29 | 0.19 | 0.01 | 6 |
| *Opuntia maxima* Mill. | 29 | 0.19 | 0.01 | 6 |
| *Equisetum arvense* L. | 28 | 0.18 | 0.01 | 14 |
| *Teucrium chamaedrys* L. | 28 | 0.18 | 0.01 | 9 |
| *Tilia platyphyllos* Scop. | 28 | 0.18 | 0.01 | 9 |
| *Agrimonia eupatoria* L. | 27 | 0.18 | 0.01 | 9 |
| *Inula viscosa* (L.) Ait. | 27 | 0.18 | 0.01 | 10 |
| *Lavandula latifolia* Medic. | 27 | 0.18 | 0.01 | 8 |
| *Lythrum salicaria* L. | 27 | 0.18 | 0.01 | 2 |
| *Sedum sediforme* (Jacq.) Pau | 27 | 0.18 | 0.01 | 9 |
| *Glycyrrhiza glabra* L. | 26 | 0.17 | 0.01 | 9 |
| *Mentha suaveolens* Ehrh. | 26 | 0.17 | 0.01 | 7 |
| *Prunus spinosa* L. | 26 | 0.17 | 0.01 | 5 |
| *Satureja montana* L. | 26 | 0.17 | 0.01 | 8 |
| *Helichrysum stoechas* (L.) Moench | 25 | 0.16 | 0.01 | 7 |
| *Tanacetum vulgare* L. | 25 | 0.16 | 0.01 | 8 |
| *Tilia* sp. | 25 | 0.16 | - | 6 |
| *Cucumis sativus* L. | 24 | 0.16 | 0.01 | 5 |
| *Althaea officinalis* L. | 23 | 0.15 | 0.01 | 8 |
| *Centaurium pulchellum* (Swartz) Druce | 23 | 0.15 | 0.01 | 4 |
| *Leuzea conifera* (L.) DC. in Lam. et DC. | 23 | 0.15 | 0.01 | 5 |
| *Mentha longifolia* (L.) Huds. | 23 | 0.15 | 0.01 | 6 |
| *Paliurus spina-christi* Mill. | 23 | 0.15 | 0.01 | 2 |
| *Spartium junceum* L. | 23 | 0.15 | 0.01 | 6 |
| *Abies alba* Mill. | 22 | 0.14 | 0.01 | 10 |
| *Ceratonia siliqua* L. | 22 | 0.14 | 0.01 | 3 |
| *Chelidonium majus* L. | 22 | 0.14 | 0.01 | 4 |
| *Cichorium intybus* L. | 22 | 0.14 | 0.01 | 10 |
| *Nepeta cataria* L. | 22 | 0.14 | 0.01 | 5 |
| *Valeriana officinalis* L. | 22 | 0.14 | 0.01 | 5 |
| *Illicium verum* Hook.f. | 21 | 0.14 | 0.01 | 4 |
| *Robinia pseudoacacia* L. | 21 | 0.14 | 0.01 | 3 |
| *Fraxinus excelsior* L. | 20 | 0.13 | 0.01 | 7 |
| *Verbena officinalis* L. | 20 | 0.13 | 0.01 | 10 |
| *Ajuga chamaepitys* (L.) Schreb. subsp. *chamaepitys* | 19 | 0.12 | 0.01 | 4 |
| *Asperula cynanchica* L. | 19 | 0.12 | 0.01 | 10 |
| *Convolvulus arvensis* L. | 19 | 0.12 | 0.01 | 8 |
| *Arctium minus* Bernh. | 18 | 0.12 | 0.01 | 7 |
| *Artemisia alba* Turra | 18 | 0.12 | 0.01 | 4 |
| *Medicago sativa* L. | 18 | 0.12 | 0.01 | 9 |
| *Phlomis lychnitis* L. | 18 | 0.12 | 0.01 | 7 |
| *Thymelaea tinctoria* (Pourr.) Endl. | 18 | 0.12 | 0.01 | 3 |
| *Clematis flammula* L. | 17 | 0.11 | 0.01 | 4 |
| *Cupressus sempervirens* L. | 17 | 0.11 | 0.01 | 5 |
| *Ocimum basilicum* L. | 17 | 0.11 | 0.01 | 7 |
| *Rubia peregrina* L. | 17 | 0.11 | 0.01 | 5 |
| *Trifolium alpinum* L. | 17 | 0.11 | 0.01 | 8 |
| *Dictamnus hispanicus* Webb ex Willk. | 16 | 0.10 | 0.01 | 6 |
| *Meum athamanticum* Jacq. subsp. *athamanticum* | 16 | 0.10 | 0.01 | 5 |
| *Quercus coccifera* L. subsp. *coccifera* | 16 | 0.10 | 0.01 | 4 |

| | | | | |
|---|---|---|---|---|
| *Betula pendula* Roth | 15 | 0.10 | 0.01 | 2 |
| *Globularia vulgaris* L. | 15 | 0.10 | 0.01 | 6 |
| *Mercurialis annua* L. | 15 | 0.10 | 0.01 | 3 |
| *Polygonum aviculare* L. | 15 | 0.10 | 0.01 | 4 |
| *Solanum tuberosum* L. | 15 | 0.10 | 0.01 | 5 |
| *Vaccinium myrtillus* L. | 15 | 0.10 | 0.01 | 2 |
| *Verbascum thapsus* L. | 15 | 0.10 | 0.01 | 7 |
| *Cichorium endivia* L. | 14 | 0.09 | 0.01 | 6 |
| *Cinnamomum verum* J.Presl | 14 | 0.09 | 0.01 | 8 |
| *Ficus carica* L. | 14 | 0.09 | 0.01 | 9 |
| *Hedera helix* L. | 14 | 0.09 | 0.01 | 9 |
| *Nicotiana tabacum* L. | 14 | 0.09 | 0.01 | 3 |
| *Rhamnus alaternus* L. | 14 | 0.09 | 0.01 | 6 |
| *Sanguisorba minor* Scop. | 14 | 0.09 | 0.01 | 7 |
| *Verbascum pulverulentum* Vill. | 14 | 0.09 | 0.01 | 5 |
| *Borago officinalis* L. | 13 | 0.09 | 0.01 | 5 |
| *Actinidia chinensis* Planch. | 12 | 0.08 | 0.01 | 1 |
| *Alchemilla alpina* L. | 12 | 0.08 | 0.01 | 5 |
| *Apium graveolens* L. | 12 | 0.08 | 0.01 | 6 |
| *Avena sativa* L. | 12 | 0.08 | 0.01 | 5 |
| *Buxus sempervirens* L. | 12 | 0.08 | 0.01 | 6 |
| *Ceterach officinarum* DC. in Lam. et DC. | 12 | 0.08 | 0.01 | 7 |
| *Lavandula angustifolia* Mill. | 12 | 0.08 | 0.01 | 6 |
| *Mentha aquatica* L. | 12 | 0.08 | 0.01 | 4 |
| *Mercurialis tomentosa* L. | 12 | 0.08 | 0.01 | 2 |
| *Ononis viscosa* L. subsp. *breviflora* (DC.) Nyman | 12 | 0.08 | 0.01 | 5 |
| *Polypodium vulgare* L. | 12 | 0.08 | 0.01 | 5 |
| *Ramonda myconi* (L.) Reichenb. | 12 | 0.08 | 0.01 | 8 |
| *Tanacetum corymbosum* (L.) Sch.Bip. | 12 | 0.08 | 0.01 | 4 |
| *Artemisia vulgaris* L. | 11 | 0.07 | 0.01 | 3 |
| *Digitalis obscura* L. | 11 | 0.07 | 0.01 | 3 |
| *Diospyros kaki* Linn.f. | 11 | 0.07 | 0.01 | 4 |
| *Filipendula ulmaria* (L.) Maxim. | 11 | 0.07 | 0.01 | 5 |
| *Hypericum ericoides* L. | 11 | 0.07 | 0.01 | 4 |
| *Piper nigrum* L. | 11 | 0.07 | 0.01 | 2 |
| *Urtica urens* L. | 11 | 0.07 | 0.01 | 7 |
| *Viola alba* Besser | 11 | 0.07 | 0.01 | 6 |
| *Citrus aurantium* L. | 10 | 0.07 | 0.00 | 3 |
| *Cynara cardunculus* L. | 10 | 0.07 | 0.00 | 4 |
| *Herniaria hirsuta* L. | 10 | 0.07 | 0.00 | 5 |
| *Polygala vulgaris* L. | 10 | 0.07 | 0.00 | 3 |
| *Prunella vulgaris* L. | 10 | 0.07 | 0.00 | 5 |
| *Salix cinerea* L. | 10 | 0.07 | 0.00 | 2 |
| *Silene saxifraga* L. | 10 | 0.07 | 0.00 | 4 |
| *Silybum marianum* (L.) Gaertn. | 10 | 0.07 | 0.00 | 2 |
| *Aristolochia pistolochia* L. | 9 | 0.06 | 0.00 | 4 |
| *Bryonia cretica* L. subsp. *dioica* (Jacq.) Tutin | 9 | 0.06 | 0.00 | 7 |
| *Cuminum cyminum* L. | 9 | 0.06 | 0.00 | 5 |
| *Equisetum telmateia* Ehrh. | 9 | 0.06 | 0.00 | 6 |
| *Pinus halepensis* Mill. | 9 | 0.06 | 0.00 | 4 |
| *Platanus ×hispanica* Mill. ex Münchh. | 9 | 0.06 | 0.00 | 3 |

| | | | | |
|---|---|---|---|---|
| *Scabiosa atropurpurea* L. | 9 | 0.06 | 0.00 | 3 |
| *Sideritis tragoriganum* Lag. | 9 | 0.06 | 0.00 | 1 |
| *Syzygium aromaticum* (L.) Merr. et Perry | 9 | 0.06 | 0.00 | 2 |
| *Thymbra capitata* (L.) Cav. | 9 | 0.06 | 0.00 | 4 |
| *Verbascum sinuatum* L. | 9 | 0.06 | 0.00 | 3 |
| *Zea mays* L. | 9 | 0.06 | 0.00 | 5 |
| *Althaea cannabina* L. subsp. *cannabina* | 8 | 0.05 | 0.00 | 2 |
| *Arbutus unedo* L. | 8 | 0.05 | 0.00 | 3 |
| *Aristolochia rotunda* L. | 8 | 0.05 | 0.00 | 2 |
| *Asplenium septentrionale* (L.) Hoffm. | 8 | 0.05 | 0.00 | 4 |
| *Olea europaea* L. subsp. *europaea* var. *sylvestris* (Mill.) Brot. | 8 | 0.05 | 0.00 | 5 |
| *Otanthus maritimus* (L.) Hoffms. et Link | 8 | 0.05 | 0.00 | 4 |
| *Phalaris canariensis* L. | 8 | 0.05 | 0.00 | 2 |
| *Prunus dulcis* (Mill.) Weeb. | 8 | 0.05 | 0.00 | 6 |
| *Pyrus communis* L. | 8 | 0.05 | 0.00 | 1 |
| *Rosa* sp. | 8 | 0.05 | - | 3 |
| *Rumex crispus* L. | 8 | 0.05 | 0.00 | 4 |
| *Rumex longifolius* DC. in Lam. et DC. | 8 | 0.05 | 0.00 | 2 |
| *Sarothamnus scoparius* (L.) Wimm. ex Koch | 8 | 0.05 | 0.00 | 5 |
| *Sideritis hirsuta* L. | 8 | 0.05 | 0.00 | 4 |
| *Smilax aspera* L. | 8 | 0.05 | 0.00 | 5 |
| *Solanum melongena* L. | 8 | 0.05 | 0.00 | 3 |
| *Stachys heraclea* All. | 8 | 0.05 | 0.00 | 6 |
| *Theobroma cacao* L. | 8 | 0.05 | 0.00 | 1 |
| *Triticum* sp. | 8 | 0.05 | - | 1 |
| *Vinca minor* L. | 8 | 0.05 | 0.00 | 1 |
| *Aloe vera* (L.) Burm.f. | 7 | 0.05 | 0.00 | 3 |
| *Calendula officinalis* L. | 7 | 0.05 | 0.00 | 5 |
| *Castanea sativa* Mill. | 7 | 0.05 | 0.00 | 1 |
| *Centaurea nigra* L. | 7 | 0.05 | 0.00 | 2 |
| *Chamaemelum nobile* (L.) All. | 7 | 0.05 | 0.00 | 5 |
| *Chenopodium ambrosioides* L. | 7 | 0.05 | 0.00 | 5 |
| *Fraxinus angustifolia* Vahl | 7 | 0.05 | 0.00 | 3 |
| *Galinsoga parviflora* Cav. | 7 | 0.05 | 0.00 | 2 |
| *Lavatera arborea* L. | 7 | 0.05 | 0.00 | 4 |
| *Mentha ×gentilis* L. | 7 | 0.05 | 0.00 | 3 |
| *Quercus humilis* Mill. | 7 | 0.05 | 0.00 | 1 |
| *Raphanus raphanistrum* L. | 7 | 0.05 | 0.00 | 5 |
| *Rosa pimpinellifolia* L. subsp. *pimpinellifolia* | 7 | 0.05 | 0.00 | 1 |
| *Ruta montana* (L.) L. | 7 | 0.05 | 0.00 | 3 |
| *Salvia pratensis* L. subsp. *pratensis* | 7 | 0.05 | 0.00 | 3 |
| *Sedum album* L. | 7 | 0.05 | 0.00 | 5 |
| *Solanum lycopersicum* L. | 7 | 0.05 | 0.00 | 3 |
| *Aesculus hippocastanum* L. | 6 | 0.04 | 0.00 | 4 |
| *Alyssum maritimum* (L.) Lam. | 6 | 0.04 | 0.00 | 3 |
| *Artemisia campestris* L. | 6 | 0.04 | 0.00 | 3 |
| *Brassica oleracea* L. subsp. *oleracea* var. *acephala* (DC.) Thell. | 6 | 0.04 | 0.00 | 3 |
| *Centaurea aspera* L. | 6 | 0.04 | 0.00 | 3 |
| *Coffea arabica* L. | 6 | 0.04 | 0.00 | 4 |
| *Cucurbita pepo* L. | 6 | 0.04 | 0.00 | 4 |
| *Ecballium elaterium* (L.) A.Richard in Bory | 6 | 0.04 | 0.00 | 3 |

| | | | | |
|---|---|---|---|---|
| *Equisetum* sp. | 6 | 0.04 | - | 6 |
| *Fragaria vesca* L. | 6 | 0.04 | 0.00 | 3 |
| *Ilex aquifolium* L. | 6 | 0.04 | 0.00 | 4 |
| *Malva nicaeensis* All. | 6 | 0.04 | 0.00 | 3 |
| *Origanum virens* Hoffms. et Link | 6 | 0.04 | 0.00 | 2 |
| *Phagnalon saxatile* (L.) Cass. | 6 | 0.04 | 0.00 | 4 |
| *Pinguicula vulgaris* L. | 6 | 0.04 | 0.00 | 1 |
| *Pinus sylvestris* L. | 6 | 0.04 | 0.00 | 3 |
| *Plantago coronopus* L. | 6 | 0.04 | 0.00 | 3 |
| *Plantago media* L. | 6 | 0.04 | 0.00 | 5 |
| *Potentilla erecta* (L.) Räuschel | 6 | 0.04 | 0.00 | 4 |
| *Rorippa nasturtium-aquaticum* (L.) Hayek | 6 | 0.04 | 0.00 | 5 |
| *Satureja hortensis* L. | 6 | 0.04 | 0.00 | 2 |
| *Sempervivum tectorum* L. | 6 | 0.04 | 0.00 | 5 |
| *Sonchus oleraceus* L. | 6 | 0.04 | 0.00 | 4 |
| *Sonchus tenerrimus* L. | 6 | 0.04 | 0.00 | 4 |
| *Symphytum tuberosum* L. subsp. *tuberosum* | 6 | 0.04 | 0.00 | 3 |
| *Verbascum* sp. | 6 | 0.04 | - | 6 |
| *Artemisia* sp. | 5 | 0.03 | - | 2 |
| *Artemisia verlotiorum* Lamotte | 5 | 0.03 | 0.00 | 2 |
| *Arum italicum* Mill. | 5 | 0.03 | 0.00 | 3 |
| *Ballota hirsuta* Benth. | 5 | 0.03 | 0.00 | 4 |
| *Ballota nigra* L. subsp. *foetida* (Vis.) Hayek | 5 | 0.03 | 0.00 | 4 |
| *Bupleurum fruticescens* L. | 5 | 0.03 | 0.00 | 3 |
| *Buxus balearica* Lam. | 5 | 0.03 | 0.00 | 2 |
| *Cassia italica* (Mill.) Lam ex Andrews | 5 | 0.03 | 0.00 | 2 |
| *Corylus avellana* L. | 5 | 0.03 | 0.00 | 2 |
| *Daphne laureola* L. | 5 | 0.03 | 0.00 | 3 |
| *Dianthus seguieri* Vill. | 5 | 0.03 | 0.00 | 2 |
| *Dorycnium pentaphyllum* Scop. | 5 | 0.03 | 0.00 | 4 |
| *Fumaria officinalis* L. | 5 | 0.03 | 0.00 | 4 |
| *Globularia cordifolia* L. | 5 | 0.03 | 0.00 | 3 |
| *Hieracium pilosella* L. | 5 | 0.03 | 0.00 | 1 |
| *Hordeum vulgare* L. | 5 | 0.03 | 0.00 | 3 |
| *Lavatera cretica* L. | 5 | 0.03 | 0.00 | 4 |
| *Myrtus communis* L. | 5 | 0.03 | 0.00 | 3 |
| *Ononis spinosa* L. | 5 | 0.03 | 0.00 | 1 |
| *Pelargonium* sp. | 5 | 0.03 | - | 3 |
| *Pinus* sp. | 5 | 0.03 | - | 3 |
| *Plantago sempervirens* Crantz | 5 | 0.03 | 0.00 | 5 |
| *Platanus orientalis* L. subsp. *acerifolia* Aiton. | 5 | 0.03 | 0.00 | 1 |
| *Potentilla reptans* L. | 5 | 0.03 | 0.00 | 5 |
| *Quercus faginea* Lam. subsp. *faginea* | 5 | 0.03 | 0.00 | 2 |
| *Rosa arvensis* Huds. | 5 | 0.03 | 0.00 | 2 |
| *Ruscus aculeatus* L. | 5 | 0.03 | 0.00 | 4 |
| *Salvia verbenaca* L. | 5 | 0.03 | 0.00 | 4 |
| *Saxifraga vayredana* Luiz. | 5 | 0.03 | 0.00 | 2 |
| *Sedum* sp. | 5 | 0.03 | - | 1 |
| *Sedum telephium* L. | 5 | 0.03 | 0.00 | 2 |
| *Solanum nigrum* L. | 5 | 0.03 | 0.00 | 3 |
| *Spinacia oleracea* L. | 5 | 0.03 | 0.00 | 1 |

| | | | | |
|---|---|---|---|---|
| *Tanacetum balsamita* L. | 5 | 0.03 | 0.00 | 2 |
| *Trifolium pratense* L. | 5 | 0.03 | 0.00 | 3 |
| *Ulmus minor* Mill. | 5 | 0.03 | 0.00 | 5 |
| *Umbilicus rupestris* (Salisb.) Dandy | 5 | 0.03 | 0.00 | 5 |
| *Vicia faba* L. | 5 | 0.03 | 0.00 | 2 |
| *Althaea rosea* (L.) Cav. | 4 | 0.03 | 0.00 | 3 |
| *Amelanchier ovalis* Medic. | 4 | 0.03 | 0.00 | 4 |
| *Aristolochia longa* L. subsp. *paucinervis* (Pomel) Batt. in Batt. et Trab. | 4 | 0.03 | 0.00 | 2 |
| *Calendula arvensis* L. | 4 | 0.03 | 0.00 | 3 |
| *Calluna vulgaris* (L.) Hull | 4 | 0.03 | 0.00 | 3 |
| *Capsella bursa-pastoris* (L.) Medic. | 4 | 0.03 | 0.00 | 3 |
| *Cephalaria leucantha* (L.) Roem. et Schultes | 4 | 0.03 | 0.00 | 2 |
| *Crocus sativus* L. | 4 | 0.03 | 0.00 | 2 |
| *Daphne gnidium* L. | 4 | 0.03 | 0.00 | 4 |
| *Daphne mezereum* L. | 4 | 0.03 | 0.00 | 1 |
| *Daucus carota* L. subsp. *carota* | 4 | 0.03 | 0.00 | 2 |
| *Dorycnium hirsutum* (L.) Ser. in DC. | 4 | 0.03 | 0.00 | 3 |
| *Epilobium angustifolium* L. | 4 | 0.03 | 0.00 | 1 |
| *Eupatorium cannabinum* L. subsp. *cannabinum* | 4 | 0.03 | 0.00 | 3 |
| *Helichrysum italicum* (Roth) G.Don f. in Loundon | 4 | 0.03 | 0.00 | 2 |
| *Mespilus germanica* L. | 4 | 0.03 | 0.00 | 1 |
| *Onobrychis viciifolia* Scop. | 4 | 0.03 | 0.00 | 3 |
| *Orobanche crenata* Forsk. | 4 | 0.03 | 0.00 | 2 |
| *Pelargonium capitatum* Ait. | 4 | 0.03 | 0.00 | 3 |
| *Plantago afra* L. | 4 | 0.03 | 0.00 | 3 |
| *Prunella grandiflora* (L.) Scholler | 4 | 0.03 | 0.00 | 4 |
| *Prunus armeniaca* L. | 4 | 0.03 | 0.00 | 2 |
| *Prunus persica* (L.) Batsch | 4 | 0.03 | 0.00 | 2 |
| *Quercus cerrioides* Willk. et Costa | 4 | 0.03 | 0.00 | 1 |
| *Quercus petraea* (Matt.) Liebl. | 4 | 0.03 | 0.00 | 1 |
| *Rosa gallica* L. | 4 | 0.03 | 0.00 | 4 |
| *Rumex* sp. | 4 | 0.03 | - | 2 |
| *Secale cereale* L. | 4 | 0.03 | 0.00 | 3 |
| *Sedum acre* L. | 4 | 0.03 | 0.00 | 2 |
| *Sedum dasyphyllum* L. | 4 | 0.03 | 0.00 | 2 |
| *Sideritis spinulosa* Barnades ex Asso | 4 | 0.03 | 0.00 | 3 |
| *Thymelaea hirsuta* (L.) Endl. | 4 | 0.03 | 0.00 | 2 |
| *Tribulus terrestris* L. | 4 | 0.03 | 0.00 | 2 |
| *Ulmus glabra* Huds. | 4 | 0.03 | 0.00 | 2 |
| *Urtica* sp. | 4 | 0.03 | - | 4 |
| *Viola odorata* L. | 4 | 0.03 | 0.00 | 4 |
| *Agave americana* L. | 3 | 0.02 | 0.00 | 2 |
| *Alkanna tinctoria* Tausch | 3 | 0.02 | 0.00 | 2 |
| *Allium porrum* L. | 3 | 0.02 | 0.00 | 2 |
| *Arnica montana* L. subsp. *montana* | 3 | 0.02 | 0.00 | 3 |
| *Artemisia arborescens* L. | 3 | 0.02 | 0.00 | 2 |
| *Arundo donax* L. | 3 | 0.02 | 0.00 | 1 |
| *Asparagus acutifolius* L. | 3 | 0.02 | 0.00 | 2 |
| *Beta vulgaris* L. subsp. *vulgaris* var. *crassa* (Alef.) Helm | 3 | 0.02 | 0.00 | 2 |
| *Brassica nigra* (L.) Koch in Roehl | 3 | 0.02 | 0.00 | 2 |

| | | | | |
|---|---|---|---|---|
| *Cinchona officinalis* L. | 3 | 0.02 | 0.00 | 1 |
| *Cistus monspeliensis* L. | 3 | 0.02 | 0.00 | 2 |
| *Cnicus benedictus* L. | 3 | 0.02 | 0.00 | 3 |
| *Conium maculatum* L. | 3 | 0.02 | 0.00 | 2 |
| *Cucurbita ficifolia* C.D.Bouché in Verh. | 3 | 0.02 | 0.00 | 3 |
| *Cynoglossum cheirifolium* L. | 3 | 0.02 | 0.00 | 2 |
| *Datura stramonium* L. | 3 | 0.02 | 0.00 | 1 |
| *Dianthus caryophyllus* L. | 3 | 0.02 | 0.00 | 2 |
| *Equisetum ramosissimum* Desf. | 3 | 0.02 | 0.00 | 3 |
| *Galium verum* L. subsp. *verum* | 3 | 0.02 | 0.00 | 3 |
| *Genista scorpius* (L.) DC. in Lam. et DC. subsp. *scorpius* | 3 | 0.02 | 0.00 | 3 |
| *Geranium robertianum* L. | 3 | 0.02 | 0.00 | 2 |
| *Helianthemum origanifolium* (Lam.) Pers. subsp. *origanifolium* | 3 | 0.02 | 0.00 | 2 |
| *Knautia arvensis* (L.) Coult. | 3 | 0.02 | 0.00 | 3 |
| *Lactuca sativa* L. | 3 | 0.02 | 0.00 | 3 |
| *Lavandula dentata* L. | 3 | 0.02 | 0.00 | 1 |
| *Lavandula* sp. | 3 | 0.02 | - | 3 |
| *Lens culinaris* Medic. subsp. *culinaris* | 3 | 0.02 | 0.00 | 1 |
| *Lepidium graminifolium* L. subsp. *graminifolium* | 3 | 0.02 | 0.00 | 1 |
| *Lepidium latifolium* L. | 3 | 0.02 | 0.00 | 3 |
| *Leucanthemum vulgare* Lam. | 3 | 0.02 | 0.00 | 1 |
| *Ligustrum vulgare* L. | 3 | 0.02 | 0.00 | 2 |
| *Matricaria aurea* (Loefl.) Schultz Bip. | 3 | 0.02 | 0.00 | 3 |
| *Morus alba* L. | 3 | 0.02 | 0.00 | 3 |
| *Myristica fragrans* Houtt. | 3 | 0.02 | 0.00 | 3 |
| *Paronychia argentea* Lam. | 3 | 0.02 | 0.00 | 3 |
| *Paronychia kapela* (Hacq.) Kerner | 3 | 0.02 | 0.00 | 3 |
| *Phaseolus vulgaris* L. | 3 | 0.02 | 0.00 | 2 |
| *Plantago* sp. | 3 | 0.02 | - | 3 |
| *Prunella laciniata* (L.) L. | 3 | 0.02 | 0.00 | 3 |
| *Psoralea bituminosa* L. | 3 | 0.02 | 0.00 | 3 |
| *Retama sphaerocarpa* (L.) Boiss. | 3 | 0.02 | 0.00 | 2 |
| *Rosa tomentosa* Sm. | 3 | 0.02 | 0.00 | 2 |
| *Senecio leucophyllus* DC. | 3 | 0.02 | 0.00 | 2 |
| *Sideritis romana* L. | 3 | 0.02 | 0.00 | 2 |
| *Sideritis* sp. | 3 | 0.02 | - | 2 |
| *Solanum dulcamara* L. | 3 | 0.02 | 0.00 | 2 |
| *Telephium imperati* L. subsp. *imperati* | 3 | 0.02 | 0.00 | 1 |
| *Trigonella foenum-graecum* L. | 3 | 0.02 | 0.00 | 3 |
| *Verbascum boerhavii* L. | 3 | 0.02 | 0.00 | 1 |
| *Veronica austriaca* L. subsp. *teucrium* (L.) D.A.Webb | 3 | 0.02 | 0.00 | 3 |
| *Vinca difformis* Pourr. | 3 | 0.02 | 0.00 | 2 |
| *Viola sylvestris* Lam. | 3 | 0.02 | 0.00 | 2 |
| *Achillea ageratum* L. | 2 | 0.01 | 0.00 | 2 |
| *Adiantum capillus-veneris* L. | 2 | 0.01 | 0.00 | 2 |
| *Alchemilla vulgaris* L. | 2 | 0.01 | 0.00 | 2 |
| *Allium ampeloprasum* L. | 2 | 0.01 | 0.00 | 2 |
| *Anacyclus clavatus* (Desf.) Pers. | 2 | 0.01 | 0.00 | 2 |
| *Ananas comosus* (Stickm.) Merr. | 2 | 0.01 | 0.00 | 2 |
| *Anethum graveolens* L. | 2 | 0.01 | 0.00 | 2 |
| *Arctostaphylos uva-ursi* (L.) Spreng. | 2 | 0.01 | 0.00 | 1 |

| | | | | |
|---|---|---|---|---|
| *Artemisia dracunculus* L. | 2 | 0.01 | 0.00 | 1 |
| *Avena barbata* Pott ex Link in Schrad. | 2 | 0.01 | 0.00 | 2 |
| *Beta vulgaris* L. subsp. *vulgaris* var. *altissima* Döll | 2 | 0.01 | 0.00 | 1 |
| *Beta vulgaris* L. subsp. *vulgaris* var. *conditiva* Alef. | 2 | 0.01 | 0.00 | 2 |
| *Brachypodium phoenicoides* (L.) Roem. et Schultes | 2 | 0.01 | 0.00 | 1 |
| *Brassica oleracea* L. subsp. *oleracea* var. *botrytis* L. | 2 | 0.01 | 0.00 | 2 |
| *Calystegia sepium* (L.) R.Br. | 2 | 0.01 | 0.00 | 1 |
| *Capsicum annuum* L. | 2 | 0.01 | 0.00 | 1 |
| *Cardamine pyrenaica* (L. in Loefl.) O.Kuntze subsp. *pyrenaica* | 2 | 0.01 | 0.00 | 1 |
| *Centaurea jacea* L. | 2 | 0.01 | 0.00 | 1 |
| *Centaurea pectinata* L. subsp. *pectinata* | 2 | 0.01 | 0.00 | 1 |
| *Centaurium spicatum* (L.) Fritsch | 2 | 0.01 | 0.00 | 1 |
| *Chenopodium album* L. | 2 | 0.01 | 0.00 | 1 |
| *Cicer arietinum* L. | 2 | 0.01 | 0.00 | 1 |
| *Cistus albidus* L. | 2 | 0.01 | 0.00 | 2 |
| *Crataegus azarolus* L. | 2 | 0.01 | 0.00 | 2 |
| *Crithmum maritimum* L. | 2 | 0.01 | 0.00 | 2 |
| *Cucumis melo* L. | 2 | 0.01 | 0.00 | 2 |
| *Cuscuta epithymum* (L.) L. | 2 | 0.01 | 0.00 | 1 |
| *Cyperus rotundus* L. | 2 | 0.01 | 0.00 | 2 |
| *Dianthus* sp. | 2 | 0.01 | - | 1 |
| *Echium vulgare* L. | 2 | 0.01 | 0.00 | 2 |
| *Eucalyptus globulus* Labill. | 2 | 0.01 | 0.00 | 2 |
| *Helianthemum marifolium* (L.) Mill. subsp. *rotundifolium* (Dunal) O.Bolòs et J.Vigo | 2 | 0.01 | 0.00 | 2 |
| *Helianthemum nummularium* (L.) Mill. | 2 | 0.01 | 0.00 | 2 |
| *Helianthemum origanifolium* (Lam.) Pers. subsp. *molle* (Cav.) F.Q. et Rothm. | 2 | 0.01 | 0.00 | 1 |
| *Helianthus tuberosus* L. | 2 | 0.01 | 0.00 | 1 |
| *Hypericum maculatum* Crantz | 2 | 0.01 | 0.00 | 1 |
| *Inula helenium* L. | 2 | 0.01 | 0.00 | 2 |
| *Iris germanica* L. | 2 | 0.01 | 0.00 | 1 |
| *Iris pseudacorus* L. | 2 | 0.01 | 0.00 | 2 |
| *Juniperus phoenicea* L. | 2 | 0.01 | 0.00 | 1 |
| *Lilium candidum* L. | 2 | 0.01 | 0.00 | 1 |
| *Malva parviflora* L. | 2 | 0.01 | 0.00 | 2 |
| *Mantisalca salmantica* (L.) Briq. et Cavill. | 2 | 0.01 | 0.00 | 1 |
| *Matricaria discoidea* DC. | 2 | 0.01 | 0.00 | 1 |
| *Morus nigra* L. | 2 | 0.01 | 0.00 | 2 |
| *Nerium oleander* L. | 2 | 0.01 | 0.00 | 1 |
| *Phlomis crinita* Cav. | 2 | 0.01 | 0.00 | 2 |
| *Phyllitis scolopendrium* (L.) Newm. | 2 | 0.01 | 0.00 | 2 |
| *Pinus mugo* Turra subsp. *uncinata* (Ramond ex DC. in Lam. et DC.) Domin | 2 | 0.01 | 0.00 | 2 |
| *Portulaca oleracea* L. | 2 | 0.01 | 0.00 | 2 |
| *Quercus pubescens* Willd. | 2 | 0.01 | 0.00 | 1 |
| *Quercus suber* L. | 2 | 0.01 | 0.00 | 1 |
| *Ranunculus bulbosus* L. | 2 | 0.01 | 0.00 | 1 |
| *Reseda alba* L. subsp. *alba* | 2 | 0.01 | 0.00 | 1 |
| *Reseda lutea* L. | 2 | 0.01 | 0.00 | 1 |
| *Rheum rhabarbarum* L. | 2 | 0.01 | 0.00 | 2 |

| | | | | |
|---|---|---|---|---|
| *Rosa micrantha* Borrer ex Sm. in Sowerby | 2 | 0.01 | 0.00 | 1 |
| *Rubus canescens* DC. | 2 | 0.01 | 0.00 | 1 |
| *Rumex conglomeratus* Murray | 2 | 0.01 | 0.00 | 1 |
| *Saccharum officinarum* L. | 2 | 0.01 | 0.00 | 1 |
| *Salsola kali* L. | 2 | 0.01 | 0.00 | 2 |
| *Satureja acinos* (L.) Scheele | 2 | 0.01 | 0.00 | 2 |
| *Satureja rouyana* Briq. | 2 | 0.01 | 0.00 | 1 |
| *Scabiosa columbaria* L. subsp. *columbaria* | 2 | 0.01 | 0.00 | 1 |
| *Senecio vulgaris* L. | 2 | 0.01 | 0.00 | 1 |
| *Sideritis scordioides* L. | 2 | 0.01 | 0.00 | 2 |
| *Stachys officinalis* (L.) Trevisan | 2 | 0.01 | 0.00 | 2 |
| *Stachys recta* L. | 2 | 0.01 | 0.00 | 1 |
| *Staehelina dubia* L. | 2 | 0.01 | 0.00 | 1 |
| *Syringa vulgaris* L. | 2 | 0.01 | 0.00 | 2 |
| *Thymus longiflorus* Boiss. subsp. *ciliatus* (Sandwith ex Lacaita) Rivas Mart. | 2 | 0.01 | 0.00 | 1 |
| *Trifolium angustifolium* L. | 2 | 0.01 | 0.00 | 2 |
| *Trifolium repens* L. | 2 | 0.01 | 0.00 | 2 |
| *Trigonella coerulea* (L.) Ser. in DC. subsp. *coerulea* | 2 | 0.01 | 0.00 | 1 |
| *Typha latifolia* L. | 2 | 0.01 | 0.00 | 1 |
| *Ulex parviflorus* Pourr. | 2 | 0.01 | 0.00 | 2 |
| *Urtica membranacea* Poiret in Lam. | 2 | 0.01 | 0.00 | 2 |
| *Verbascum lychnitis* L. | 2 | 0.01 | 0.00 | 1 |
| *Veronica officinalis* L. | 2 | 0.01 | 0.00 | 2 |
| *Xanthium strumarium* L. | 2 | 0.01 | 0.00 | 2 |
| *Ziziphus jujuba* Mill. | 2 | 0.01 | 0.00 | 1 |
| *Acanthus mollis* L. | 1 | 0.01 | 0.00 | 1 |
| *Achillea odorata* L. | 1 | 0.01 | 0.00 | 1 |
| *Aconitum napellus* L. | 1 | 0.01 | 0.00 | 1 |
| *Alnus glutinosa* (L.) Gaertn. | 1 | 0.01 | 0.00 | 1 |
| *Aloe prolifera* Haw. | 1 | 0.01 | 0.00 | 1 |
| *Aloe saponaria* Haw. | 1 | 0.01 | 0.00 | 1 |
| *Alyssum spinosum* L. | 1 | 0.01 | 0.00 | 1 |
| *Amaranthus* sp. | 1 | 0.01 | - | 1 |
| *Anacyclus valentinus* L. | 1 | 0.01 | 0.00 | 1 |
| *Anchusa italica* Retz. | 1 | 0.01 | 0.00 | 1 |
| *Anthemis arvensis* L. | 1 | 0.01 | 0.00 | 1 |
| *Anthyllis cytisoides* L. | 1 | 0.01 | 0.00 | 1 |
| *Antirrhinum barrelieri* Boreau | 1 | 0.01 | 0.00 | 1 |
| *Apium nodiflorum* (L.) Lag. subsp. *nodiflorum* | 1 | 0.01 | 0.00 | 1 |
| *Arachis hypogaea* L. | 1 | 0.01 | 0.00 | 1 |
| *Artemisia abrotanum* L. | 1 | 0.01 | 0.00 | 1 |
| *Artemisia chamaemelifolia* Vill. | 1 | 0.01 | 0.00 | 1 |
| *Asparagus officinalis* L. | 1 | 0.01 | 0.00 | 1 |
| *Asplenium adiantum-nigrum* L. | 1 | 0.01 | 0.00 | 1 |
| *Asplenium trichomanes* L. | 1 | 0.01 | 0.00 | 1 |
| *Atropa belladonna* L. | 1 | 0.01 | 0.00 | 1 |
| *Berberis vulgaris* L. | 1 | 0.01 | 0.00 | 1 |
| *Botrychium lunaria* (L.) Swartz | 1 | 0.01 | 0.00 | 1 |
| *Brachypodium retusum* (Pers.) Beauv. | 1 | 0.01 | 0.00 | 1 |
| *Brassica napus* L. | 1 | 0.01 | 0.00 | 1 |

| | | | | |
|---|---|---|---|---|
| *Bupleurum rigidum* L. | 1 | 0.01 | 0.00 | 1 |
| *Camellia japonica* L. | 1 | 0.01 | 0.00 | 1 |
| *Camellia sinensis* L. | 1 | 0.01 | 0.00 | 1 |
| *Cannabis sativa* L. | 1 | 0.01 | 0.00 | 1 |
| *Capparis spinosa* L. | 1 | 0.01 | 0.00 | 1 |
| *Carduus tenuiflorus* Curtis | 1 | 0.01 | 0.00 | 1 |
| *Carlina acaulis* L. | 1 | 0.01 | 0.00 | 1 |
| *Centaurea calcitrapa* L. | 1 | 0.01 | 0.00 | 1 |
| *Centaurea cyanus* L. | 1 | 0.01 | 0.00 | 1 |
| *Chamaerops humilis* L. | 1 | 0.01 | 0.00 | 1 |
| *Chondrilla juncea* L. | 1 | 0.01 | 0.00 | 1 |
| *Cirsium arvense* (L.) Scop. | 1 | 0.01 | 0.00 | 1 |
| *Citrus deliciosa* Ten. | 1 | 0.01 | 0.00 | 1 |
| *Cneorum tricoccon* L. | 1 | 0.01 | 0.00 | 1 |
| *Colutea arborescens* L. | 1 | 0.01 | 0.00 | 1 |
| *Convolvulus* sp. | 1 | 0.01 | - | 1 |
| *Crepis vesicaria* L. | 1 | 0.01 | 0.00 | 1 |
| *Croton* sp. | 1 | 0.01 | - | 1 |
| *Cucurbita maxima* Duch. in Lam. | 1 | 0.01 | 0.00 | 1 |
| *Cymbopogon citratus* Stapf | 1 | 0.01 | 0.00 | 1 |
| *Cynoglossum creticum* Mill. | 1 | 0.01 | 0.00 | 1 |
| *Descurainia sophia* (L.) Webb ex Prantl in Engl. et Prantl | 1 | 0.01 | 0.00 | 1 |
| *Dictamnus albus* L. | 1 | 0.01 | 0.00 | 1 |
| *Dipsacus fullonum* L. | 1 | 0.01 | 0.00 | 1 |
| *Dorycnium rectum* (L.) Ser. in DC. | 1 | 0.01 | 0.00 | 1 |
| *Echinops ritro* L. | 1 | 0.01 | 0.00 | 1 |
| *Echium creticum* L. | 1 | 0.01 | 0.00 | 1 |
| *Erica arborea* L. | 1 | 0.01 | 0.00 | 1 |
| *Erica multiflora* L. | 1 | 0.01 | 0.00 | 1 |
| *Erica scoparia* L. subsp. *scoparia* | 1 | 0.01 | 0.00 | 1 |
| *Erigeron acer* L. | 1 | 0.01 | 0.00 | 1 |
| *Erodium cicutarium* (L.) L'Hér. in Ait. | 1 | 0.01 | 0.00 | 1 |
| *Erodium malacoides* (L.) L'Hér. | 1 | 0.01 | 0.00 | 1 |
| *Eruca vesicaria* (L.) Cav. | 1 | 0.01 | 0.00 | 1 |
| *Fraxinus ornus* L. | 1 | 0.01 | 0.00 | 1 |
| *Fumaria parviflora* Lam. | 1 | 0.01 | 0.00 | 1 |
| *Galium aparine* L. | 1 | 0.01 | 0.00 | 1 |
| *Genista balansae* (Boiss.) Rouy subsp. *europaea* (G.López et Ch.E.Jarvis) O.Bolòs et J.Vigo | 1 | 0.01 | 0.00 | 1 |
| *Gentiana verna* L. | 1 | 0.01 | 0.00 | 1 |
| *Geum urbanum* L. | 1 | 0.01 | 0.00 | 1 |
| *Gomphocarpus fruticosus* (L.) Ait.f. in Ait. | 1 | 0.01 | 0.00 | 1 |
| *Hedera algeriensis* Hibberd | 1 | 0.01 | 0.00 | 1 |
| *Hedysarum confertum* Desf. | 1 | 0.01 | 0.00 | 1 |
| *Helianthemum* sp. | 1 | 0.01 | - | 1 |
| *Helianthemum syriacum* (Jacq.) Dum.Cours. | 1 | 0.01 | 0.00 | 1 |
| *Helianthus annuus* L. | 1 | 0.01 | 0.00 | 1 |
| *Helleborus foetidus* L. | 1 | 0.01 | 0.00 | 1 |
| *Hyparrhenia hirta* (L.) Stapf in Oliver | 1 | 0.01 | 0.00 | 1 |
| *Ipomoea batatas* Poir. | 1 | 0.01 | 0.00 | 1 |
| *Lactuca virosa* L. | 1 | 0.01 | 0.00 | 1 |

| | | | | |
|---|---|---|---|---|
| *Lamium album* L. | 1 | 0.01 | 0.00 | 1 |
| *Lamium flexuosum* Ten. | 1 | 0.01 | 0.00 | 1 |
| *Lathyrus tuberosus* L. | 1 | 0.01 | 0.00 | 1 |
| *Lepidium campestre* (L.) R.Br. | 1 | 0.01 | 0.00 | 1 |
| *Linaria cymbalaria* (L.) Mill. | 1 | 0.01 | 0.00 | 1 |
| *Linum tenuifolium* L. subsp. *suffruticosum* (L.) Litard. | 1 | 0.01 | 0.00 | 1 |
| *Lithospermum fruticosum* L. | 1 | 0.01 | 0.00 | 1 |
| *Lonicera implexa* Ait. | 1 | 0.01 | 0.00 | 1 |
| *Lonicera periclymenum* L. | 1 | 0.01 | 0.00 | 1 |
| *Lycopus europaeus* L. | 1 | 0.01 | 0.00 | 1 |
| *Magnolia grandiflora* L. | 1 | 0.01 | 0.00 | 1 |
| *Marrubium supinum* L. | 1 | 0.01 | 0.00 | 1 |
| *Matthiola fruticulosa* (L.) Maire in Jah. et Maire | 1 | 0.01 | 0.00 | 1 |
| *Mentha arvensis* L. | 1 | 0.01 | 0.00 | 1 |
| *Moehringia muscosa* L. | 1 | 0.01 | 0.00 | 1 |
| *Monarda didyma* L. | 1 | 0.01 | 0.00 | 1 |
| *Musa* sp. | 1 | 0.01 | - | 1 |
| *Musa ×paradisiaca* L. | 1 | 0.01 | 0.00 | 1 |
| *Myoporum laetum* G.Forst. | 1 | 0.01 | 0.00 | 1 |
| *Opuntia vulgaris* Mill. | 1 | 0.01 | 0.00 | 1 |
| *Oxalis pes-caprae* L. | 1 | 0.01 | 0.00 | 1 |
| *Parietaria lusitanica* L. | 1 | 0.01 | 0.00 | 1 |
| *Paronychia capitata* (L.) Lam. | 1 | 0.01 | 0.00 | 1 |
| *Passiflora caerulea* L. | 1 | 0.01 | 0.00 | 1 |
| *Pelargonium odoratissimum* (L.) L'Hér. | 1 | 0.01 | 0.00 | 1 |
| *Pelargonium peltatum* (L.) L'Hér. | 1 | 0.01 | 0.00 | 1 |
| *Persea gratissima* Gaertn.f. | 1 | 0.01 | 0.00 | 1 |
| *Phagnalon sordidum* (L.) Reichenb. | 1 | 0.01 | 0.00 | 1 |
| *Phillyrea angustifolia* L. | 1 | 0.01 | 0.00 | 1 |
| *Phleum phleoides* (L.) Karsten | 1 | 0.01 | 0.00 | 1 |
| *Pimpinella tragium* Vill. | 1 | 0.01 | 0.00 | 1 |
| *Pinus pinea* L. | 1 | 0.01 | 0.00 | 1 |
| *Plantago albicans* L. | 1 | 0.01 | 0.00 | 1 |
| *Plantago lagopus* L. | 1 | 0.01 | 0.00 | 1 |
| *Plantago ovata* Forsk. | 1 | 0.01 | 0.00 | 1 |
| *Polygonum bistorta* L. | 1 | 0.01 | 0.00 | 1 |
| *Primula acaulis* (L.) L. | 1 | 0.01 | 0.00 | 1 |
| *Prunus cerasifera* Ehrh. | 1 | 0.01 | 0.00 | 1 |
| *Prunus cerasus* L. | 1 | 0.01 | 0.00 | 1 |
| *Pulicaria dysenterica* (L.) Bernh. | 1 | 0.01 | 0.00 | 1 |
| *Ranunculus ficaria* L. | 1 | 0.01 | 0.00 | 1 |
| *Reichardia picroides* (L.) Roth | 1 | 0.01 | 0.00 | 1 |
| *Rhamnus frangula* L. | 1 | 0.01 | 0.00 | 1 |
| *Rhamnus lycioides* L. subsp. *oleoides* (L.) Jah. et Maire | 1 | 0.01 | 0.00 | 1 |
| *Rheum palmatum* L. | 1 | 0.01 | 0.00 | 1 |
| *Ribes rubrum* L. | 1 | 0.01 | 0.00 | 1 |
| *Rubus caesius* L. | 1 | 0.01 | 0.00 | 1 |
| *Rumex acetosa* L. | 1 | 0.01 | 0.00 | 1 |
| *Rumex acetosella* L. | 1 | 0.01 | 0.00 | 1 |
| *Rumex pseudoalpinus* Höfft | 1 | 0.01 | 0.00 | 1 |
| *Rumex pulcher* L. | 1 | 0.01 | 0.00 | 1 |

| | | | | |
|---|---|---|---|---|
| *Salvia sclarea* L. | 1 | 0.01 | 0.00 | 1 |
| *Sanguisorba officinalis* L. | 1 | 0.01 | 0.00 | 1 |
| *Saponaria officinalis* L. | 1 | 0.01 | 0.00 | 1 |
| *Satureja graeca* L. | 1 | 0.01 | 0.00 | 1 |
| *Satureja intricata* Lange subsp. *gracilis* (Willk.) R.Morales, G.López and Sánchez-Gómez | 1 | 0.01 | 0.00 | 1 |
| *Saxifraga paniculata* Mill. | 1 | 0.01 | 0.00 | 1 |
| *Sedum brevifolium* DC. | 1 | 0.01 | 0.00 | 1 |
| *Senecio doria* L. | 1 | 0.01 | 0.00 | 1 |
| *Silene vulgaris* (Moench) Garcke | 1 | 0.01 | 0.00 | 1 |
| *Sinapis alba* L. | 1 | 0.01 | 0.00 | 1 |
| *Sisymbrium irio* L. | 1 | 0.01 | 0.00 | 1 |
| *Solidago virgaurea* L. | 1 | 0.01 | 0.00 | 1 |
| *Sorbus aria* (L.) Crantz | 1 | 0.01 | 0.00 | 1 |
| *Sorghum halepense* (L.) Pers. | 1 | 0.01 | 0.00 | 1 |
| *Stellaria media* (L.) Vill. | 1 | 0.01 | 0.00 | 1 |
| *Stipa tenacissima* L. | 1 | 0.01 | 0.00 | 1 |
| *Taraxacum dissectum* (Ledeb.) Ledeb. | 1 | 0.01 | 0.00 | 1 |
| *Taxus baccata* L. | 1 | 0.01 | 0.00 | 1 |
| *Teucrium expassum* Pau | 1 | 0.01 | 0.00 | 1 |
| *Teucrium flavum* L. subsp. *glaucum* (Jord. et Fourr.) Ronniger | 1 | 0.01 | 0.00 | 1 |
| *Teucrium pseudochamaepitys* L. | 1 | 0.01 | 0.00 | 1 |
| *Thymus* sp. | 1 | 0.01 | - | 1 |
| *Tilia cordata* Mill. | 1 | 0.01 | 0.00 | 1 |
| *Trifolium incarnatum* L. subsp. *incarnatum* | 1 | 0.01 | 0.00 | 1 |
| *Tussilago farfara* L. | 1 | 0.01 | 0.00 | 1 |
| *Urtica pilulifera* L. | 1 | 0.01 | 0.00 | 1 |
| *Verbena chamaedryfolia* Juss. | 1 | 0.01 | 0.00 | 1 |
| *Vincetoxicum hirundinaria* Medic. subsp. *intermedium* (Loret et Barr.) Markgraf | 1 | 0.01 | 0.00 | 1 |
| *Viola tricolor* L. | 1 | 0.01 | 0.00 | 1 |
| *Viscum album* L. | 1 | 0.01 | 0.00 | 1 |
| *Zingiber officinale* Roscoe | 1 | 0.01 | 0.00 | 1 |

**Table S2.** A representation of the 46 uses, providing details on the number of use reports (UR) and their corresponding percentages for each use, along with the total associated taxa and the medicinal importance (MI) index.

| Uses | UR | UR (%) | Total Taxa | MI |
|---|---|---|---|---|
| Digestive | 2652 | 17.62 | 218 | 12.17 |
| Intestinal anti-inflammatory | 2361 | 15.68 | 41 | 57.59 |
| Antidiarrheal | 1342 | 8.91 | 91 | 14.75 |
| For toothache | 1038 | 6.90 | 197 | 5.27 |
| Laxative | 957 | 6.36 | 141 | 6.79 |
| Orexigenic | 906 | 6.02 | 111 | 8.16 |
| Hepatoprotective | 771 | 5.12 | 51 | 15.12 |
| Buccal antiseptic | 716 | 4.76 | 52 | 13.77 |
| Stomachic | 647 | 4.30 | 192 | 3.37 |
| Gastric anti-inflammatory | 521 | 3.46 | 102 | 5.11 |
| Hypolipemic | 440 | 2.92 | 139 | 3.17 |
| Purgative | 377 | 2.50 | 154 | 2.45 |
| Hepatic anti-inflammatory | 325 | 2.16 | 95 | 3.42 |
| Carminative | 294 | 1.95 | 66 | 4.45 |
| Antinauseous | 270 | 1.79 | 17 | 15.88 |
| For gastrointestinal disorders | 164 | 1.09 | 7 | 23.43 |
| Buccal anti-inflammatory | 144 | 0.96 | 33 | 4.36 |
| Antiulcerous | 130 | 0.86 | 35 | 3.71 |
| Hypouricemic | 124 | 0.82 | 73 | 1.70 |
| Refrigerant | 92 | 0.61 | 46 | 2.00 |
| Antiemetic | 75 | 0.50 | 14 | 5.36 |
| Anti-icteric | 69 | 0.46 | 16 | 4.31 |
| For dental reinforcement | 58 | 0.39 | 22 | 2.64 |
| Vitaminic | 57 | 0.38 | 10 | 5.70 |
| Diaphoretic | 54 | 0.36 | 8 | 6.75 |
| Emetic | 52 | 0.35 | 18 | 2.89 |
| Antihalitosic | 43 | 0.29 | 69 | 0.62 |
| For obesity treatment | 39 | 0.26 | 7 | 5.57 |
| Gingival anti-inflammatory | 37 | 0.25 | 9 | 4.11 |
| Antacid | 35 | 0.23 | 26 | 1.35 |
| For dyspepsia | 33 | 0.22 | 85 | 0.39 |
| Hepatic lithotriptic | 32 | 0.21 | 45 | 0.71 |
| Biliary lithotriptic | 28 | 0.19 | 27 | 1.04 |
| For colic | 27 | 0.18 | 38 | 0.71 |
| Gingival antiseptic | 26 | 0.17 | 13 | 2.00 |
| For metabolic disorders | 26 | 0.17 | 20 | 1.30 |
| For gallbladder inflammation | 15 | 0.10 | 12 | 1.25 |
| Antidiaphoretic | 14 | 0.09 | 12 | 1.17 |
| Gastric/intestinal emollient | 14 | 0.09 | 19 | 0.74 |
| For appendicitis | 12 | 0.08 | 51 | 0.24 |
| Antiscorbutic | 12 | 0.08 | 22 | 0.55 |
| Sialagogic | 9 | 0.06 | 11 | 0.82 |
| For iron deficiency | 8 | 0.05 | 5 | 1.60 |
| Antioxidant | 5 | 0.03 | 3 | 1.67 |
| Antisialagogic | 2 | 0.01 | 1 | 2.00 |
| For gaining weight | 1 | 0.01 | 1 | 1.00 |
